# Optimal design of fractional-order proportional integral derivative controllers for structural vibration suppression

**DOI:** 10.1038/s41598-024-68281-2

**Published:** 2024-07-26

**Authors:** Saeed Khodadoost, Meysam Saraee, Siamak Talatahari, Pooya Sareh

**Affiliations:** 1 Department of Civil Engineering, University of Tabriz, Tabriz, Iran; 2 School of Computing, Macquarie University, Sydney, Australia; 3 Department of Computer Science, Khazar University, Baku, Azerbaijan; 4 School of Engineering, University of Liverpool, Liverpool, UK; 5 School of Engineering, Newcastle University, Newcastle Upon Tyne, UK; 6 Escuela Técnica Superior de Ingeniería y Diseño Industrial, Universidad Politécnica de Madrid, Madrid, Spain

**Keywords:** Design optimization, Fractional calculus, FOPID controller, Structural vibration, Earthquake, Statistical test, Civil engineering, Engineering, Mechanical engineering

## Abstract

In designing control systems, it is known that fractional-order proportional integral derivative (FOPID) controllers often provide greater flexibility than conventional proportional integral derivative (PID) controllers. This higher level of flexibility has proven to be extremely valuable for various applications such as vibration suppression in structural engineering. In this paper, we study the optimization of FOPID controllers using twelve well-established algorithms to minimize structural responses under seismic excitations. The algorithms include crystal structure algorithm (CryStAl), stochastic paint optimizer, particle swarm optimization, krill herd, harmony search, ant colony optimization, genetic algorithm, grey wolf optimizer, Harris hawks optimization, sparrow search algorithm, hippopotamus optimization algorithm, and duck swarm algorithm. In addition to highlighting the benefits of fractional calculus in structural control, this study provides a detailed analysis of FOPID controllers as well as a brief description of the algorithms used to optimize them. To evaluate the efficiency of the proposed techniques, two building models with different numbers of stories are examined. FOPID controllers are designed based on oustaloup’s approximation and the El Centro earthquake data. Using five well-known metrics, the performances of the developed methods are evaluated against five earthquake scenarios, including the recent earthquake in Turkey. A non-parametric (Friedman) test is also employed to compare the algorithms based on their corresponding vibration reduction. The findings of this analysis show that CryStAl consistently performs better than the other algorithms for both building models, thus resulting in superior vibration suppression.

## Introduction

The application of fractional calculus has been proven to be highly useful for the precise modeling of various engineering systems and structures. In particular, fractional derivatives and integrals have been shown to be more effective than their integer-order counterparts in a wide range of engineering processes. Xu et al.^1^ studied the complex structure of fractional viscoelastic beams by using the wave propagation method. They showed that the utilization of the Riemann–Liouville fractional calculus is a suitable approach for characterizing the properties of viscoelastic materials. Using Haar wavelet analysis, Khodadoost and Katebi^2^ presented methods for solving fractional order differential equations in the context of engineering structures. Their results showed strong agreement with the existing literature, demonstrating their potential for implementation in practical applications.

The general objective of control in structural systems is to modify the performance features of the systems to achieve the desired response^3–10^. Controllers that employ fractional-order calculus are generally robust against uncertainties of various kinds which may arise from both modeling and evaluation procedures. The presence of uncertainties can affect the optimal performance of control systems. Recently, fractional calculus has found extensive applications in diverse areas of science and engineering; these include the exploration of fractional-order controllers^11–13^, mechanical vibrations^14,15^, and fractional systems^16–18^.

Proportional–integral–derivative (PID) controllers are widely used in various industrial control systems. Combining fractional calculus with PID controllers results in fractional-order PID, known as FOPID, controllers. There are different types of tuning methods for FOPID controllers, including metaheuristic algorithms; more specifically, a broad range of algorithms has been used to optimize FOPID controllers including the genetic algorithm (GA), harmony search (HS), ant colony optimization (ACO), particle swarm optimization (PSO), krill herd (KH), and gray wolf optimizer (GWO)^19–23^.

Yong and Ren^24^ utilized FOPID controllers optimized by the PSO and artificial bee colony (ABC) algorithms and implemented them in both fractional-order and integer-order systems. This study demonstrated that PSO yielded better responses and greater robustness. In another study by Ibrahim et al.^25^, PSO results were compared with those of the ACO algorithm for the speed control of a DC motor, leading to the conclusion that PSO performed better in terms of frequency response but worse in terms of maximum overshoot. Nasir and Khadraoui^26^ showed that GA outperformed PSO in finding optimized coefficients for FOPID controllers. Overall, PSO has been used in numerous previous studies, with the results being compared to conventional approaches or other metaheuristic algorithms. It can be inferred that, depending on the system and controllers used, it could be one of the most effective choices^27–29^.

Mohamed et al.^30^ investigated the potential of FOPID controllers to improve frequency variation and power control in a microgrid power plant. They utilized the KH algorithm for parameter optimization of the FOPID controller and conducted a comparative analysis with the results obtained using GA. In another study, Mohamed et al.^31^ compared KH and HS results to optimize a power system FOPID controller. Roy et al.^32^ utilized the differential harmony search (DHS) algorithm to optimize a FOPID controller and compared the results with those of GA and PSO, concluding that DHS was a more suitable choice. Ramadhas^33^ proposed and optimized a FOPID controller Harmony Search, showing its advantages over conventional controllers in regulating the speed of a DC motor when subjected to heavy load conditions. Mughees and Mohsin ^34^ developed a magnetic levitation system using FOPID controllers optimized by the ACO algorithm. The results of a comparative study demonstrated that the ACO-based controllers exhibited higher efficiency than the conventional Ziegler-Nichols technique. Similarly, Chiranjeevi^35^ utilized GA and ACO to design FOPID controllers for automatic voltage regulator (AVR) systems, finding that GA outperformed the ACO algorithm.

Cu et al.^42^ introduced the Improved Sparrow Search Algorithm (IMSSA), an algorithm for optimizing parameters in FOPID controllers. IMSSA outperformed other algorithms, including SSA and GWO, in terms of speed, accuracy, and robustness, offering a novel approach to solving control problems efficiently. Chen et al.^36^ presented an Improved SSA (ISSA) aimed at optimizing parameter tuning for FOPID controllers. Testing on ten benchmark functions demonstrated that ISSA enhanced convergence accuracy, speed, and global search capabilities. Additionally, simulations of two control systems validated the optimization and effectiveness of parameter tuning for FOPID controllers using ISSA when compared to existing results. Jaballah et al.^37^ introduced an optimal active vibration control system utilizing an active tuned mass damper (ATMD) to mitigate seismic structural damages from earthquakes. The proposed controller incorporated fractional-order calculus to enhance the performance of the conventional PID controller. The parameters of the FOPID controller were optimized using the Artificial Hummingbird Algorithm (AHA). Comparative studies against classical PID controllers were conducted under various earthquake excitations to demonstrate the advantages of the proposed FOPID^37^. Verma^38^ presented a novel evolutionary technique for optimizing fractional-order controller parameters. The GWO algorithm was employed for tuning both integer- and fractional-order controllers. Performance indices, such as integral square error and integral absolute error, were minimized to validate the effectiveness of the proposed approach. The algorithm was further validated and compared with established techniques.

In the area of control systems, it has been commonplace to use hybrid algorithms to achieve improved optimal results. Oladipo et al.^39^ utilized the Hybrid Particle Swarm and Grey Wolf Optimization (HPSGWO) algorithm to optimize FOPID controllers. Comparisons were made with the PSO and GWO algorithms, proving satisfactory results. Komathi et al.^40^ proposed a GWO-based fractional-order proportional-integral (FOPI) controller for the power factor correction of conventional proportional-integral (PI) controllers. FOPI controllers offered better robustness and stability due to an additional adjustable parameter.

The Stochastic Paint Optimizer (SPO) algorithm is a recent metaheuristic algorithm taking inspiration from the art of painting^41^. Khodadadi et al.^42^ presented a multi-objective version of this algorithm called MOSPO and compared its results with other multi-objective algorithms, showing competitive performance for MOSPO.

Talatahari et al.^43–45^ introduced CryStAl and evaluated it against 239 mathematical functions, comparing it to tweleve other metaheuristic algorithms. The results demonstrated its competitive performance and superiority in most cases, supported by statistical analyses. Farooqui et al.^46^ utilized CryStAl to solve nonlinear transcendental equations. CryStAl was used to solve the equations involved in determining the optimum switching angles, where the effectiveness of CryStAl was demonstrated through comparisons with other metaheuristic algorithms. In another study, Wang et al. ^47^ proposed an improved crystal structure algorithm; they employed the correlation between the golden sine function and the unit circle to expand the scope of the algorithm’s search space. The optimization of gable frames with tapered members was performed by Salama et al.^48^ through the utilization of the enhanced crystal structure algorithm (ECryStAl). The results were compared with those obtained from different algorithms, demonstrating the overall high performance of ECryStAl. The algorithm CryStAl was also employed to design optimized fuzzy controllers that intelligently managed vibrations induced by seismic activity in an active control system^49^. The algorithm's performance was demonstrated using two real-size building structures, one with three stories and the other with twenty stories. The Crystal Structure Algorithm consistently outperformed other algorithms in most cases.

Table 1 compares previous research on the performance of various optimization algorithms in different scenarios. It can be seen from this table that while various optimization techniques have been applied to the design of FOPID controllers^58–69^, the potential of more recent algorithms such as SPO, CryStAl, hippopotamus optimization (HO), and duck swarm algorithm (DSA) for this purpose has not been explored, leading to a gap in the literature in this specific context. This research aims to address this issue by investigating the performance of FOPID controllers optimized using such recent algorithms. By comparing their results, we will present a comparative study on the effectiveness of these techniques in enhancing FOPID controllers’ performance, particularly for seismic mitigation applications.

**Table 1:** A summary of various algorithms used in the design of FOPID controllers.

Ref	Year	Algorithms	Application	Main findings
^27^	2006	Enhanced PSO	General	An enhanced PSO algorithm for designing FOPID controllers showed improved control performance
^32^	2010	DHS	Not specified	DHS-based design of FOPID controllers led to better results compared to established optimization techniques like PSO and GA
^29^	2014	PSO	Stable, unstable, and non-minimum phase systems	PSO-optimized FOPID controllers demonstrated improved performance over conventional PID controllers in various systems
^35^	2016	GA and ACO	Automatic voltage regulator (AVR) systems	A FOPID controller was used to improve the stability and dynamic response of an AVR system, and was compared with a PID controller for transient response and robustness
^38^	2017	GWO	Time-delay and higher-order systems	GWO was used to optimize the parameters of the FOPID controller and the results were compared with those of established techniques
^50^	2018	GWO	DC motor speed control	Comparative and robustness analyses of a GWO-based FOPID controller for the speed control of DC motors were conducted
^28^	2018	PSO and ABC	Unstable and integrating systems	ABC-based controllers show advantages over PSO-based controllers in terms of PID and FOPID tuning in unstable and integrating systems
^33^	2018	HS	PEMFC powered vehicles	HS-FPID controllers used for speed regulation in PEMFC vehicles turned out to be superior to existing controllers, implemented in MATLAB Simulink
^51^	2018	Gases brownian motion optimization (GBMO)	Vibration mitigation of seismic-excited structures	The GBMO algorithm was employed for the optimal tuning of the FOPID controller parameters, and was compared with PID, LQR, and FLC controllers for seismic control
^52^	2019	GA	Fractional-order systems	A GA-based optimization of an ant colony controller was proposed for fractional-order systems
^53^	2019	PSO	Single conical tank systems	The PSO-optimized FOPID controller demonstrated better set point tracking and smoother response compared to conventional controllers
^40^	2019	GWO	Power factor correction in SMPS applications	The GWO algorithm used for FOPI controller optimization demonstrated improved performance compared to conventional PI controllers
^54^	2019	IBA	MIMO distillation column process	A DBA-based FOPID controller used to control the distillate and bottom mole fractions outperformed other optimization techniques
^30^	2020	KH	Microgrid systems	The Krill Herd algorithm outperformed GA in tuning FOPID controller parameters for microgrid frequency control
^34^	2020	ACO	Magnetic levitation systems	A FOPID controller optimized for stability control outperformed the conventional PID controller, implemented in MATLAB Simulink
^39^	2020	HPSGWO	AVR systems	The HPSGWO algorithm proposed for FOPID controller optimization demonstrated superior performance compared to PSO and GWO algorithms
^55^	2020	Ant lion optimizer (ALO)	First-order and higher-order systems	The ALO algorithm used for the tuning of FOPID controllers demonstrated superiority in transient and frequency responses
^20^	2021	N/A	Fractional-order control	This study reviewed milestones and future perspectives for the industrialization of fractional-order control
^26^	2021	PSO and GA	DC motor control	FOPID controllers for controlling DC motors were designed using PSO and GA optimization methods
^31^	2021	KH	Renewable power systems control	FOPID controller tuned by the Krill Herd algorithm enhanced stability performance compared to PID controllers in renewable power systems
^56^	2021	Gradient-based optimization (GBO)	AVR systems	The GBO algorithm used for optimal FOPID controller design showed improved dynamic response and stability
^57^	2022	PSO, GA, and ACO	PID/FOPID controller tuning	This study reviewed various metaheuristic techniques for tuning the parameters of PID/FOPID controllers
^24^	2022	PSO	Inverted pendulum control	The control performance of a fractional-order PID controller based on the PSO algorithm in an inverted pendulum system was investigated
^25^	2022	PSO and ACO	DC motor speed control	A speed controller for a DC motor based on FOPID parameters was designed using metaheuristic algorithms
^58^	2022	IMSSA	Not specified	IMSSA was proposed for the optimal tuning of FOPID controller parameters, demonstrating superior effectiveness compared to other algorithms
^36^	2022	IMSSA	Not specified	ISSA was proposed for FOPID controller parameters tuning, demonstrating improved convergence accuracy and speed
^44^	2022	AHA	Structural vibration control	A FOPID controller optimized for seismic structural damage reduction was compared with a classical PID controller
^59^	2022	Modified grey wolf optimizer (mGWO)	AVR systems	The mGWO algorithm was proposed for FOPID controller optimization, demonstrating robustness and improved convergence compared to state-of-the-art techniques
^60^	2022	Distance and levy-flight based crow search algorithm (DLCSA)	AVR systems	The DLCSA algorithm, used for the optimization of FOPID, FOPI, and FOPD controllers, demonstrated better performance compared to other techniques
^61^	2023	PSO and GA	Quadrotor control	The performances of conventional and fractional order PID controllers tuned with PSO and GA for quadrotor control were compared
^21^	2023	Multi-objective GA	High-speed elevator vibration control	An optimized FOPID method to suppress the vibrations of high-speed elevators was proposed
^62^	2023	GBMO	Time-delayed MIMO seismic-excited structural systems	The GBMO algorithm, utilized for optimal FOPID controller design, demonstrated superior performance and robustness compared to the PID controller
^63^	2023	Fractional-order actor-critic algorithm (FOAC)	Nonlinear systems	The FOPID-FOAC controller proposed for nonlinear systems showed effectiveness in tracking and stabilization
^19^	2024	PSO	Wind turbine power systems	This study applied a fractional-order synergetic-proportional integral controller based on the PSO algorithm to improve the output power of wind turbine power systems

This paper aims to identify improvement strategies and their impact on the performance of algorithms used for optimizing FOPID controllers. To this end, we first present a brief introduction to twelve different algorithms, including CryStAl, SPO, HO, and DSA, which have not been used before for the optimization of FOPID controllers. Then, we demonstrate the application of these algorithms in the design of FOPID controllers, where two building models with different numbers of stories are examined. Using a set of well-established metrics, the performances of the developed methods are evaluated against five earthquake scenarios. Finally, the best algorithm for each application is identified, and a detailed discussion of the results is provided, followed by the concluding remarks.

## An overview of FOPID controllers and their applications

**Figure 1:**
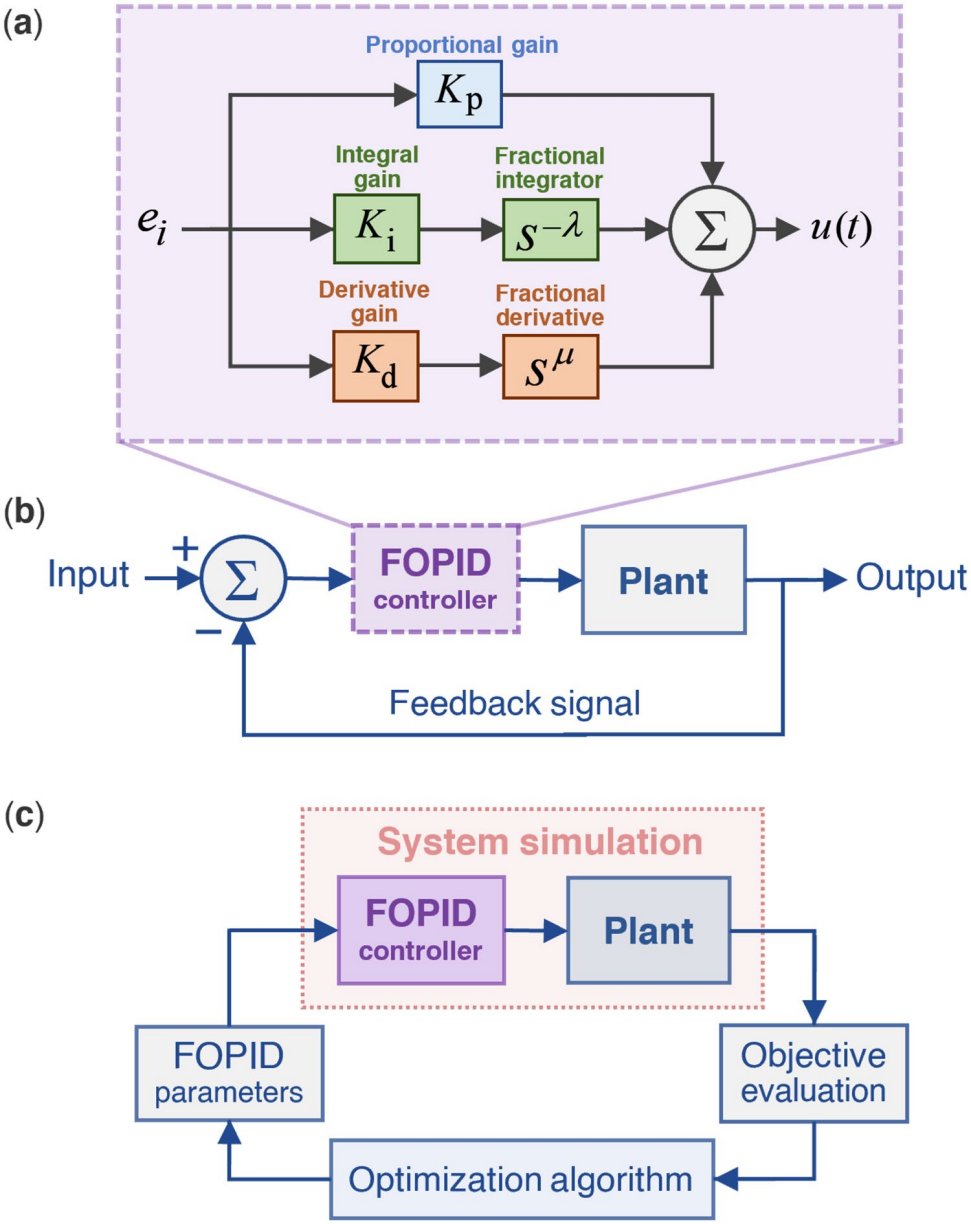
(**a**) Block diagram of a FOPID controller. (**b**) A typical closed-loop control system with a FOPID controller. (**c**) Optimization process of a FOPID controller.

The FOPID controller is a variant of the conventional PID controller that incorporates two additional parameters the selection of which is important for enhancing the overall performance of the system. The FOPID controller includes five parameters ${K}_{\text{p}},{K}_{\text{i}}$, ${K}_{\text{d}}$, $\lambda$, and $\mu$, which denote proportional gain, integral gain, derivative gain, integration order, and derivation order, respectively. Figure 1a represents the layout of a FOPID controller, in which the error signal $u\left(t\right)$ is generated by multiplying the input signal ${e}_{i}$ by ${K}_{\text{p}}$, ${K}_{\text{i}}{s}^{-\lambda }$, and ${K}_{\text{d}}{s}^{\mu }$, followed by summing up the results. Figure 1b shows a typical closed-loop control system with a FOPID controller, while (Fig. 1c) depicts the standard configuration for FOPID controller optimization. The FOPID controller’s parameters are randomly chosen using optimization techniques like metaheuristic algorithms. Subsequently, the results are evaluated, enabling designers to select the most appropriate controller.

FOPID controllers are typically used to enhance the overall performance of a system. In general, they reduce the vulnerability of variations to the parameters of a controlled system as well as the controllers themselves. The generic transfer function of a FOPID controller can be expressed as

1 $$C\left( s \right) = \frac{U\left( s \right)}{{E\left( s \right)}} = K_{{\text{p}}} + \frac{{K_{{\text{i}}} }}{{s^{\lambda } }} + K_{d} s^{\mu }$$

where $C\left(s\right)$, $U\left(s\right)$, and $E\left(s\right)$ represent controller output, control signal, and control error.

Since fractional-order operators have indefinite dimensions, integer-order approximations are used to implement FOPID controllers. Standard transfer function approximation techniques include the Oustaloup method^64^, expressed as

2 $$G\left( s \right) = s^{\alpha } \quad \left( {0 < \alpha < 1} \right)$$

The oustaloup filter can be designed using

3 $$H_{N} \left( s \right) = k\mathop \prod \limits_{n = 1}^{N} \frac{{s + \omega_{l} \omega_{u}^{{\left( {2n - 1 - \alpha } \right)/N}} }}{{s + \omega_{l} \omega_{u}^{{\left( {2n - 1 + \alpha } \right)/N}} }}$$

where

4 $$\omega_{u} = \sqrt {\frac{{\omega_{h} }}{{\omega_{b} }}} {\text{ }}{\text{ and }} {\text{ }}k = \omega_{h}^{\alpha }$$

In Eqs. (2)–(4), $\alpha$ is the order of the fractional function, $N$ is the order of the approximation, and ${\omega }_{l}$ and ${\omega }_{h}$ are the lower and higher values of the frequency range, respectively.

The Oustaloup method approximates the fractional-order differentiator by representing it as a product of a series of stable first-order linear systems. While a wide band of approximation is generally desirable because the approximation performs the best in the interior of the frequency interval, certain margins should be maintained to avoid boundary-related issues.

## Optimization algorithms

This section describes the various optimization algorithms used in this study. More recently proposed algorithms, including CryStAl and SPO, are explained in more detail with accompanying flowcharts. We recommend referring to the provided references for further information on the older algorithms.

### Particle swarm optimization (PSO)

Particle swarm optimization (PSO) is an optimization technique that takes inspiration from the cooperative behavior of groups of fish and birds. It searches a given space for the best solution to a problem by moving a collection of particles (i.e., possible solutions), where each particle has a position and velocity. The algorithm starts with an initial population of particles randomly distributed in the search area. Based on the objective function of the problem, each particle is evaluated in terms of fitness. The obtained fitness value is used to update the position and velocity of the particle, which are adjusted based on its own best position and the best position that has been discovered so far by all other particles in the swarm^65^.

As particles move through the search space, they converge toward promising regions where good solutions are likely to be found. When a stopping requirement—such as reaching the maximum number of repetitions or the target level of accuracy—is satisfied, the algorithm ends. It has been shown that many types of optimization problems, including controller design, mechanical design, image processing, data mining, and machine learning can be effectively solved using PSO^65^.

### Ant colony optimization (ACO)

Ant colony optimization (ACO) is a metaheuristic algorithm that takes inspiration from how ants forage for food. This algorithm uses a set of artificial ants to explore the problem space and find the optimal solution. The ants deposit pheromones on their travel paths, which attract other ants to follow the same path. Over time, the paths with higher pheromone levels become more attractive to the ants, leading to a convergence towards the optimal response^66^.

ACO has been used to solve a diverse range of optimization problems, including routing, scheduling, and clustering. It is particularly useful for solving problems with complex search spaces and multiple objectives. ACO has also been extended to include additional features such as local search and hybridization with other algorithms.

### Harmony search (HS)

The harmony search (HS) metaheuristic algorithm was inspired by the concept of harmony in music. In HS, a solution is represented as a set of decision variables, and each decision variable is considered a musical note. Harmonies are the initial population of solutions used by the HS algorithm. These harmonies are randomly generated within the search space. The algorithm then iteratively improves these harmonies by creating new ones through a process called improvisation. During improvisation, new harmonies are created by combining elements from existing harmonies in the population^67^.

The process of creating new harmonies involves three main steps, namely, pitch adjustment, pitch memory consideration, and randomization. In pitch adjustment, some decision variables in the new harmony are adjusted to improve its fitness value. In pitch memory consideration, some decision variables are chosen from the best harmonies in the population to enhance diversity and avoid premature convergence. Finally, randomization introduces randomness into the new harmony to explore unexplored regions of the search space. The HS algorithm continues to improve the population of harmonies until a stopping criterion is satisfied; the stopping criterion can achieve a good fitness value or the maximum number of iterations^68^.

### Krill herd (KH)

The Krill Herd (KH) algorithm simulates the social behavior of krill in a swarm, where each krill adjusts its movement and behavior based on the position and behavior of its neighbors. In the KH algorithm, each krill represents a possible solution to the optimization problem, where the algorithm identifies the optimum solution by iteratively changing the locations and behaviors of the krill. The algorithm uses several mechanisms to simulate the social behavior of krill, including attraction, repulsion, and alignment. Attraction refers to the tendency of krill to move towards areas with higher food concentrations (i.e., good solutions). Repulsion refers to the tendency of krill to avoid areas with low food concentrations (i.e., poor solutions). Alignment refers to the tendency of krill to align their movements with those of their neighbors.

The KH algorithm has efficiently solved numerous optimization problems, such as picture segmentation, function optimization, and feature selection. It has also been used in various applications in the areas of robotics, finance, and engineering design^69^.

### Genetic algorithm (GA)

The genetic algorithm (GA) is a type of evolutionary algorithm used in computing to find optimal solutions to various complex problems across a diverse range of areas. Genetic algorithms utilize principles inspired by evolutionary biology, including inheritance, mutation, selection, and crossover^70^. The GA methodology encompasses several fundamental steps. First is the initialization phase where a population of solutions is randomly generated, typically comprising hundreds to thousands of possibilities covering the entire search space. Occasionally, solutions may be strategically seeded in areas where optimal solutions are more likely to be found.

Following initialization is the ‘selection’ stage. During each successive generation, a portion of the existing population is chosen to breed the next generation. This selection process is based on fitness, with fitter solutions being more likely to be selected. Various selection methods, such as roulette wheel selection and tournament selection, are employed to maintain population diversity. After ‘selection’ comes ‘reproduction’, where selected individuals undergo genetic operations such as crossover and mutation to produce new offspring. These offspring inherit characteristics from their parents and continue the process until a new population of suitable size is generated.

Finally, the process continues until a termination condition is met. This could include finding a satisfactory solution, reaching a predetermined number of generations, exhausting computational resources, or plateauing in fitness improvement. Pseudocode for a simple genetic algorithm involves initializing a population, evaluating fitness, and iteratively selecting, breeding, and replacing individuals until termination conditions are satisfied^70^.

### Gray wolf optimizer (GWO)

Inspired by the hunting tactics of wolf packs, the gray wolf optimizer (GWO) was developed to tackle complex optimization problems^71^. Unlike algorithms requiring extensive memory, GWO operates efficiently, saving only the top three solutions. Its mathematical model effectively mimics wolf hunting behavior, balancing exploration and exploitation to avoid getting stuck in local optima. Imagine a group of ‘wolves’ representing potential solutions, forming a hierarchy with alpha, beta, delta, and omega roles. These wolves then embark on a hunt, with the alpha and beta leading the pack toward promising areas based on fitness evaluations. Delta wolves support the leaders, while omega wolves follow along. As the hunt progresses, wolves adjust their positions, encircling the ‘prey’ (optimal solution) based on the alpha and beta's locations. They strategically balance ‘attacking’ (getting closer) with ‘encircling’ (maintaining search space) behaviors, refining their solutions with each movement.

Throughout the hunt, wolves share information on their positions and fitness values, guiding the pack collectively toward the best solution. This iterative hunting process continuously improves solutions until the desired fitness level is achieved or other stopping criteria are met. By mimicking the real-world dynamics of wolf packs, GWO effectively tackles diverse optimization problems, offering users a valuable tool to explore complex environments to discover optimal solutions^72,73^.

### Harris hawks optimization (HHO)

The Harris Hawk, renowned for its cooperative hunting tactics, is the source of inspiration behind the development of the harris hawks optimization (HHO) algorithm^74^. The exploration phase of HHO parallels the initial exploration undertaken by the Hawks, where each member of the population explores the search space for optimal solutions. This phase integrates both the exploitation of the best current solution and random exploration to ensure comprehensive coverage of the solution scape. Subsequently, upon identifying a target, HHO transitions into an exploitation phase, mirroring the rapid and coordinated maneuvers employed by the hawks during hunting. This phase encompasses four distinct strategies: surprise attack, soft blockade, hard blockade, and levy flight. Through the adept utilization of these strategies, HHO effectively balances the exploration of diverse solution spaces with the exploitation of promising options, thereby progressively refining its search toward optimal solutions^74,75^.

### Sparrow search algorithm (SSA)

The Sparrow Search Algorithm (SSA) is inspired by the foraging behaviors of sparrows, particularly their adaptability and group dynamics. Sparrows are categorized as either producers or scroungers based on their foraging strategies^76^. Producers actively seek out food sources, while scroungers adopt a more observant approach, trailing behind and opportunistically capitalizing on their findings. This dichotomy between producers and scroungers serves as a foundational principle within SSA^76^.

In its implementation, SSA emulates the foraging behavior of sparrows by initiating with individuals representing potential solutions to optimization problems, akin to sparrows scouring their environment for sustenance. Producers, representing individuals with higher fitness values indicative of successful foraging, assume leadership roles within the population, guiding the direction of the search toward promising regions. Conversely, scroungers, embodying individuals with comparatively lower fitness values, trail behind the producers, benefiting from their discoveries.

Moreover, SSA integrates a mechanism similar to the vigilance exhibited by sparrows in response to perceived threats. When individuals detect danger, symbolized by high ‘alarm’ values in the algorithm, producers guide the population toward safety, preventing entrapment in local optima and ensuring continued progress in the search process^77^.

### Duck swarm algorithm (DSA)

The duck swarm algorithm (DSA) is an optimization method inspired by the foraging behavior of ducks^78^. Initially, ducks disperse randomly across the search area. Subsequently, they engage in alternating phases of exploration and exploitation. During exploration, ducks navigate based on cues from a leading duck and their immediate peers, expanding their search to identify promising zones. Upon discovering sufficient food sources, ducks transition to exploitation mode, concentrating their efforts around the most lucrative areas. In this phase, adjustments to the control parameter and cooperation/competition coefficients enable following the lead duck and successful companions, refining the search around the most favorable solutions uncovered thus far. By effectively managing the balance between exploration and exploitation, DSA endeavors to efficiently pinpoint optimal solutions within the search space^78^.

### Hippopotamus optimization (HO) algorithm

The hippopotamus optimization algorithm (HO) is a recently introduced bio-inspired algorithm inspired by the behavior of hippopotamuses^79^. Drawing inspiration from the social interactions, movement patterns, and defense mechanisms of hippopotamuses, HO finds solutions to optimization problems through a three-phased approach. The first phase concerns exploration, mimicking how hippopotamuses move within their herd and search for food sources. The second phase focuses on exploitation, similar to how hippopotamuses defend themselves against predators. Finally, the third phase incorporates local search, reflecting how hippopotamuses flee toward water bodies for safety. This combination of exploration, exploitation, and local search allows HO to effectively navigate the search space and find optimal solutions^79^.

### Stochastic paint optimizer (SPO)

The stochastic paint optimizer (SPO) is an optimization method that models the search space as a painting canvas. It utilizes the principles of color theory, where the algorithm explores and exploits the search space by generating new colors (solutions) based on existing ones^41^.

**Figure 2:**
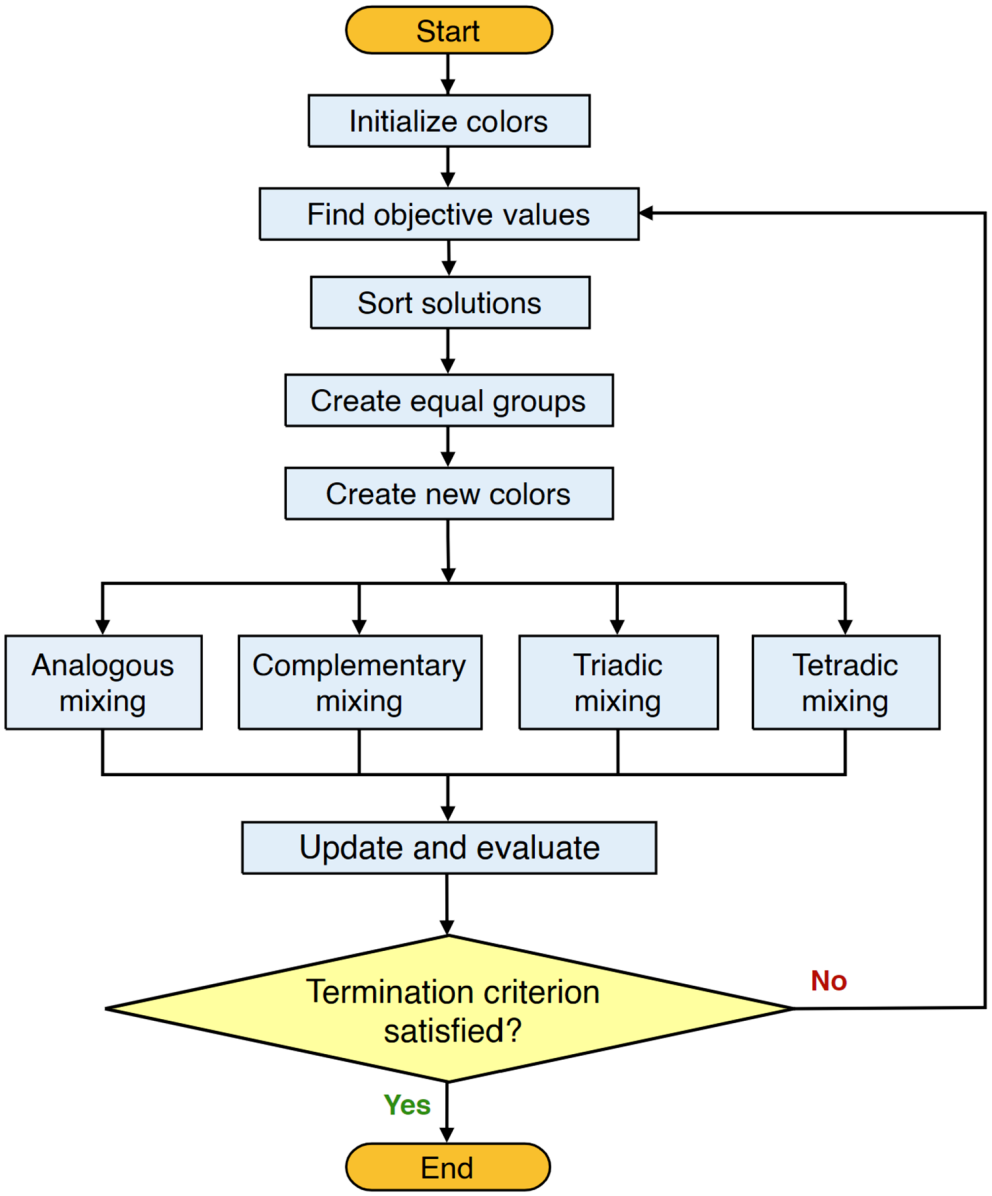
Flowchart of the stochastic paint optimizer (SPO) algorithm (adapted from ^41^).

As can be seen from Fig. 2, the algorithm incorporates four different color combination techniques: analogous, complementary, triadic, and tetradic. Each technique combines colors from different categories (primary, secondary, and tertiary) to create a new color. The analogous technique selects three neighboring colors on the color wheel as ${C}_{i-1}$, ${C}_{i}$, and ${C}_{i+1}$, and combines them as formulated in Eq. (5). On the other hand, the complementary technique combines two opposite colors, e.g. the best and the worst colors. The primary category ${C}_{\text{P},i}$, tertiary category ${C}_{\text{T},i}$, and existing color ${C}_{i}$ are combined according to Eq. (6). to generate a new color. The triadic technique uses the mean of one color from each category (i.e., primary ${C}_{\text{P},i}$, secondary ${C}_{\text{S},i}$, and tertiary ${C}_{\text{T},i}$), and the main color, to generate a new color as expressed by Eq. (7). Finally, the tetradic technique combines four colors, including a randomly generated color ${C}_{\mathfrak{R}}$ and the three colors defined by the previous equation to generate a new color according to Eq. (8).

5 $$C_{{\text{new}},1} = C_{i} + \Re \left( {C_{i + 1} - C_{i - 1} } \right)$$

6 $$C_{{\text{new}},2} = C_{i} + \Re \left({C_{{\text{P,}}i} - C_{{\text{T,}}i}} \right)$$

7 $$C_{{\text{new}},3} = C_{i} + \Re \left( {\frac{{C_{{\text{P,}}i} + C_{{\text{S,}}i} + C_{{\text{T,}}i} }}{3}} \right)$$

8 $$C_{{\text{new}},4} = C_{i} + \left( {\frac{{\Re_{1} C_{{\text{P,}}i} + \Re_{2} C_{{\text{T,}}i} + \Re_{3} C_{{\text{S,}}i} + \Re_{4} C_{\Re } }}{4}} \right)$$

In the equations above, $\mathfrak{R}$ is a random number within the range [0, 1]. The combination techniques in SPO are designed to balance exploration and exploitation. In the initial iterations, the algorithm focuses on exploring the search space by searching for better solutions near the existing ones. As the iterations progress, it shifts towards exploiting the best solutions and refining them further.

### Crystal structure algorithm (CryStAl)

**Figure 3:**
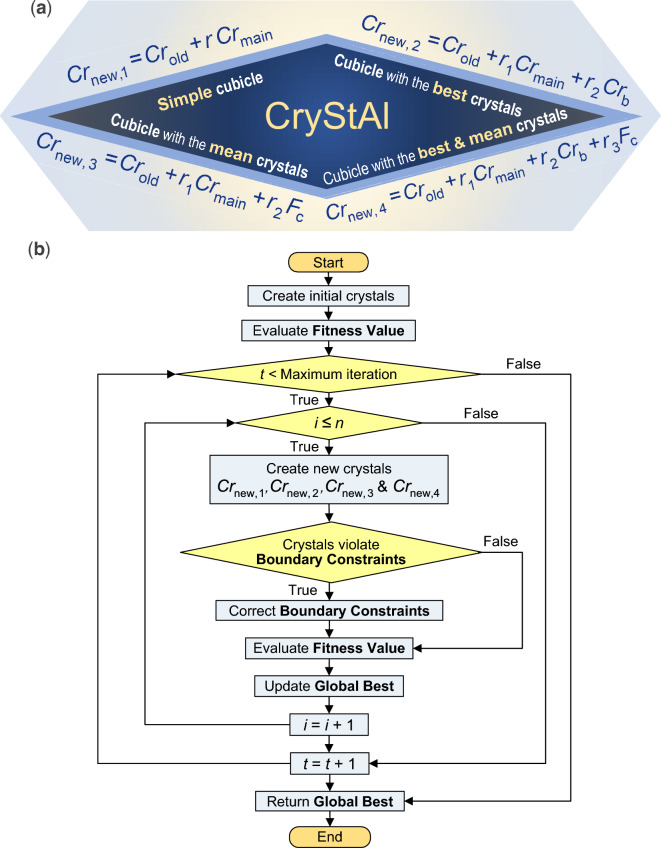
Crystal structure algorithm (CryStAl). (**a**) Four types of updating processes. (**b**) Flowchart.

Crystals are solid minerals with regular arrangements of molecules, atoms, or ions in three-dimensional space. The algorithm called CryStAl^43^, short for crystal structure algorithm, is a metaheuristic optimization method inspired by the geometric structure of crystals. The algorithm utilizes the concepts of lattice and basis in crystallography to represent candidate solutions in the optimization process. It begins by randomly initializing a set of crystals, where each crystal represents a candidate solution $C{r}_{k}=[{x}_{k}^{1} {x}_{k}^{2} . . . {x}_{k}^{j}. . . {x}_{k}^{d}]$. The positions of the crystals in the search space are determined using random numbers within the allowable variable ranges as follows:

9 $$Cr = \left[ {\begin{array}{*{20}c} {\begin{array}{*{20}c} {Cr_{1} } \\ {Cr_{2} } \\ \vdots \\ \end{array} } \\ {\begin{array}{*{20}c} {Cr_{k} } \\ \vdots \\ {Cr_{n} } \\ \end{array} } \\ \end{array} } \right] = \left[ {\begin{array}{*{20}c} {x_{1}^{1} } & {\begin{array}{*{20}c} {x_{1}^{2} } & \cdots & {\begin{array}{*{20}c} {x_{1}^{j} } & \cdots & {x_{1}^{d} } \\ \end{array} } \\ \end{array} } \\ {\begin{array}{*{20}c} {x_{2}^{1} } \\ \vdots \\ {\begin{array}{*{20}c} {x_{k}^{1} } \\ \vdots \\ {x_{n}^{1} } \\ \end{array} } \\ \end{array} } & {\begin{array}{*{20}c} {\begin{array}{*{20}c} {x_{2}^{2} } \\ \vdots \\ {\begin{array}{*{20}c} {x_{k}^{2} } \\ \vdots \\ {x_{n}^{2} } \\ \end{array} } \\ \end{array} } & {\begin{array}{*{20}c} \cdots \\ \vdots \\ {\begin{array}{*{20}c} \cdots \\ \vdots \\ \cdots \\ \end{array} } \\ \end{array} } & {\begin{array}{*{20}c} {\begin{array}{*{20}c} {x_{2}^{j} } & \ldots & {x_{2}^{d} } \\ \end{array} } \\ {\begin{array}{*{20}c} \vdots & \vdots & \vdots \\ \end{array} } \\ {\begin{array}{*{20}c} {\begin{array}{*{20}c} {x_{k}^{j} } \\ \vdots \\ {x_{n}^{j} } \\ \end{array} } & {\begin{array}{*{20}c} \cdots \\ \vdots \\ \cdots \\ \end{array} } & {\begin{array}{*{20}c} {x_{k}^{d} } \\ \vdots \\ {x_{n}^{d} } \\ \end{array} } \\ \end{array} } \\ \end{array} } \\ \end{array} } \\ \end{array} } \right] , \left\{ {\begin{array}{*{20}c} {k = 1,2,3, \ldots ,n} \\ {j = 1,2,3, \ldots ,d} \\ \end{array} } \right.$$

where *n* is the number of crystals and *d* is the dimension of the problem. The algorithm then proceeds with updating the crystal positions using crystalline lattice principles. To this end, four types of updating processes are considered: simple cubicles, cubicles with the best crystals, cubicles with the mean crystals, and cubicles with the best and mean crystals, as depicted in (Fig. 3a). In these equations, the crystals at the corners are considered the ‘main’ crystals denoted by $C{r}_{\text{main}}$, the ‘best’ crystal is represented by ${Cr}_{\text{b}}$, and the mean value of randomly selected crystals is denoted by ${F}_{\text{c}}$. Moreover, ${Cr}_{\text{new}}$ and ${Cr}_{\text{old}}$ are the new and old positions, respectively, and $r$, ${r}_{1}$, ${r}_{2}$, and ${r}_{3}$ are random numbers.

By incorporating these updating processes, the CryStAl algorithm performs both exploration and exploitation in the search space. It conducts local and global searches simultaneously, aiming to find the optimal solution. A mathematical flag is introduced to handle solution variables that violate the boundary conditions. The algorithm terminates after a fixed number of iterations.

One notable advantage of the CryStAl algorithm is its simplicity, as it does not require tuning of internal or external parameters. It generally finds viable solutions to optimization problems without being prone to convergence issues or local optima traps. The algorithm leverages the principles of crystal structure to guide the search process and explore the solution space effectively^43^. Figure 3b illustrates the flowchart of the CryStAl algorithm.

## Case study

**Figure 4:**
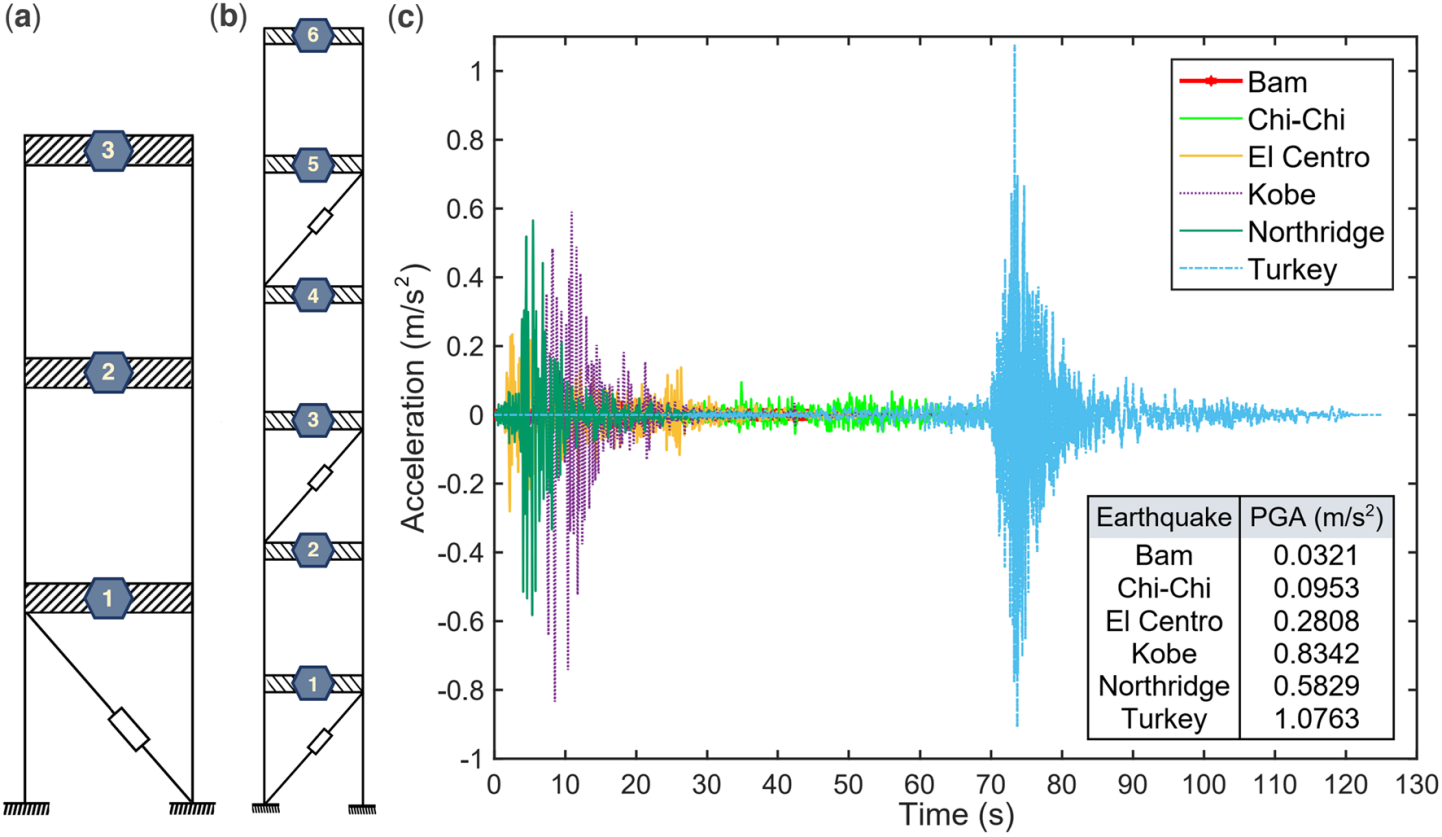
(**a**) Model of the 3-story structure. (**b**) Model of the 6-story structure. (**c**) Accelerograms of different earthquakes considered in this study.

In this section, we analyze two structures as case studies. The first structure, depicted in Fig. 4a, consists of an active tendon system placed on the first floor of a 3-story shear model building ^80^. The second structure, illustrated in (Fig. 4b), features three active tendon systems placed on the first, third, and fifth floors of a 6-story shear model building^81^. The structural properties of these two systems are summarized in (Table 2), which includes the mass, stiffness, and damping characteristics across different floors. These structures will be analyzed using six earthquake records, using a similar approach to the one demonstrated in the initial example, the earthquakes of El Centro, Turkey (Kahramanmaras Pazarck), Kobe, Bam, Northridge, and Chi-Chi are used to examine the behavior of the structures under them. The earthquakes’ parameters are listed in (Table 3), and the accelerogram is depicted in (Fig. 4c). The earthquake records are scaled to 0.112 g.

**Table 2:** Structural characteristics of the 3- and 6-story buildings.

Structure	Floor	Mass ($\text{kg})$	Stiffness ($\text{kN}/\text{m})$	Damping ($\text{kNs}/\text{m})$
3-story	1,2,3	1000	980	1.407
6-story	1	6800	231.5	7.45
	2	5897	33732	67
	3	5897	29093	58
	4	5897	28261	57
	5	5897	24954	50
	6	5897	19059	38

**Table 3:** Peak ground acceleration (PGA) of different earthquakes.

Earthquake	PGA (m/s^2^)
El Centro	0.2808
Turkey	1.0763
Chi-Chi	0.0953
Bam	0.0321
Northridge	0.5829
Kobe	0.8342

Table 4 summarizes five performance indices that are used to evaluate the simulation results. The first index, ${J}_{1}$, includes using the matching uncontrolled values to normalize the top floor’s maximum displacement. Similar to ${J}_{1}$, index ${J}_{2}$ represents the ratio of the top floor’s acceleration to that of the controlled structure. The root mean square (RMS) displacement and acceleration of the top floor in the controlled structure, as contrasted to the uncontrolled structure, are represented by indices ${J}_{3}$ and ${J}_{4}$, respectively. Finally, the ratio of the absolute maximum control force to structural weight is denoted by index ${J}_{5}$.

**Table 4:** Performance indices.

Index	Expression
Peak top-story displacement	$J_{1} = \frac{{max_{t} \left| {\left| {x_{c} \left( t \right)} \right|} \right|}}{{max_{t} \left| {\left| {x_{u} \left( t \right)} \right|} \right|}}$
Peak top-story acceleration	$J_{2} = \frac{{max_{t} \left| {\left| {a_{c} \left( t \right)} \right|} \right|}}{{max_{t} \left| {\left| {a_{u} \left( t \right)} \right|} \right|}}$
RMS top-story displacement	$J_{3} = \frac{{max_{t} \left| {\left| {\sigma_{r} \left( t \right)} \right|} \right|}}{{max_{t} \left| {\left| {\sigma_{u} \left( t \right)} \right|} \right|}}$
RMS top-story acceleration	$J_{4} = \frac{{max_{t} \left| {\left| {\partial_{r} \left( t \right)} \right|} \right|}}{{max_{t} \left| {\left| {\partial_{u} \left( t \right)} \right|} \right|}}$
Control force	$J_{5} = \frac{{max_{t} \left| {\left| {U\left( t \right)} \right|} \right|}}{W}$

To achieve an effective optimization process, the objective function must ensure the application of an appropriate level of control force, while the controller itself should exhibit robustness. Robustness implies that the controller’s performance remains stable and unaffected by uncertainties. To evaluate the sensitivity of the modeling error, a widely adopted criterion known as maximum sensitivity can be employed. Finding the reverse of the shortest distance between the critical point and the Nyquist curve of the open-loop transfer function is necessary to calculate the maximum sensitivity (${M}_{\text{s}}$). According to^82^, the following equation can be used to perform this computation:

10 $$M_{{\text{s}}} = max{ }\left( {\left| {S\left( {j\omega } \right)} \right|} \right) = max\left( {\left| {\frac{1}{{1 + g\left( {j\omega } \right)k\left( {j\omega } \right)}}} \right|} \right){ }, {\text{ }} 0 \le \omega \le \infty$$

Typically, ${M}_{\text{s}}$ remains below 2; nevertheless, it is understood that compromises must be made concerning control objectives. Lower values of ${M}_{\text{s}}$ contribute to enhanced robustness, whereas higher values result in faster response times. Thus, to strike a balance between these control objectives, an ${M}_{\text{s}}$ a value of 1.5 is selected. Furthermore, in this investigation, the parameters of the PID controller are determined by formulating the objective function, as follows

11 $$\left\{ {\begin{array}{*{20}c} {Min{\text{ }} I = \mathop \sum \limits_{i = 1}^{5} \omega_{i} J_{i} } \\{{\text{s.t.}} {\text{ }}{\text{ }} M_{{\text{s}}} \le 1.5 } \\ \end{array} } \right.$$

where ${\omega }_{1}$ = 5, ${\omega }_{2}$= 5, ${\omega }_{3}$ = 100, ${\omega }_{4}$ = 100, and ${\omega }_{5}$ = 5, and the parameters of the FOPID controller are selected randomly using various optimization algorithms^83^. Table 5 provides the list of adjustment parameters and a brief description of each algorithm.

**Table 5:** Parameters and brief description of the various optimization algorithms.

Algorithm	Parameters	Description	Value
CryStAl	Number of initial crystals	Initial population size	30
ACO	Archive size	Stores high-performing solutions	30
	Sample size	Number of solutions considered for ant movement	10
	Intensification factor	Controls the exploitation emphasis	0.5
	Deviation-distance ratio	Balances solution diversity and convergence	0.1
PSO	Swarm size	Number of particles in the swarm	30
	Inertia weight damping ratio	Controls exploration-exploitation balance	1
	Personal learning coefficient	Weights individual learning	2
	Global learning coefficient	Weights swarm-based learning	2
HS	Harmony memory size	Stores promising solutions	30
	Harmony memory consideration rate	Balances exploration and exploitation	0.95
	Pitch adjustment rate	Fine-tunes generated solutions	0.3
KH	Number of krill	Population size	50
	Visual radius	Perception range for food and other krill	0.02
	Inertia weight	Exploration-exploitation balance	0.7
	Food attraction coefficient	Strength of food attraction	1
	Herd attraction coefficient	Strength of herd attraction	0.5
	Random movement coefficient	Level of random exploration	0.5
SPO	Number of colors	Population size	30
GA	Population size	Number of individuals	30
	Crossover percentage	Rate of information exchange	0.8
	Mutation percentage	Rate of random changes	0.1
	Selection procedure	Method for choosing parents	Roulette wheel selection
GWO	Number of search agents	Population size	30
HHO	Number of search agents	Population size	30
	Exploration probability	Balances exploration and exploitation	0.5
SSA	P-percent	Proportion of producers	0.2
	Population size	Total number of sparrows	30
	Number of producers	Sparrows discovering new food sources	6
	Number of scroungers	Sparrows exploiting discovered food sources	24
	Number of scouts	Sparrows sent for random exploration	1
DSA	Population number	Population size	30
	CF_1_ and KF_1_	Cooperation coefficients	Random value in (0,2)
	CF_2_ and KF_2_	Competition coefficients	Random value in (0,2)
	FP	Scaling factor	0.618
	P	Searching conversion probability	0.5
HO	Number of search agents	Population size	30

## Results and discussion

Each algorithm was run 30 times, with the number of iterations set to 100 under El Centro earthquake. The coefficients for all FOPID controllers, which were the average values derived from the 30 runs, are summarized for each optimization method in (Table 6). After being designed based on the El Centro earthquake, the controller design was evaluated using the other five earthquakes. This strategy tried to demonstrate the controllers' effectiveness and practicality.

**Table 6:** Coefficients of FOPID controllers optimized using different algorithms.

Algorithm	3-story structure					6-story structure				
	${k}_{\text{p}}$	${k}_{\text{i}}$	${k}_{\text{d}}$	$\lambda$	$\mu$	${k}_{\text{p}}$	${k}_{\text{i}}$	${k}_{\text{d}}$	$\lambda$	$\mu$
CryStAl	7.604	−3.945	9.829	0.994	0.533	0.062	0.638	9.979	0.986	0.991
ACO	5.946	1.455	6.674	0.701	0.385	−9.875	9.915	9.892	0.899	0.001
PSO	5.550	−2.715	8.548	0.941	0.485	−1.761	8.834	9.509	0.7189	0.095
HS	2.092	0.079	8.274	0.935	0.537	−9.789	9.960	9.676	0.856	0.275
KH	3.537	−1.911	7.484	0.737	0.777	3.784	8.267	9.238	0.775	0.911
SPO	6.552	6.863	7.988	0.941	0.625	3.994	7.902	8.242	0.852	0.097
GA	6.887	−1.339	9.207	0.926	0.443	5.155	3.594	9.143	0.792	0.800
GWO	7.420	−4.392	8.528	0.895	0.537	6.090	2.499	9.972	0.992	0.173
HHO	8.547	8.644	9.637	0.924	0.901	3.004	9.884	9.951	0.903	0.003
SSA	−6.844	−7.674	8.128	0.850	0.870	−5.761	8.558	9.996	0.707	0.761
HO	7.221	9.105	8.200	0.900	0.253	8.340	3.924	8.468	0.852	0.760
DSA	8.423	9.087	8.748	0.952	0.934	8.466	8.500	8.731	0.940	0.908

These controllers were optimized to provide the best results considering the five performance indices ${J}_{1}$ to ${J}_{5}$ introduced earlier. Table 7 presents the obtained values of the indices using the various algorithms under different earthquakes.

**Table 7:** Performance indices of various algorithms under different earthquakes.

Earthquake	Algorithm	3-story structure					6-story structure				
		${J}_{1}$	${J}_{2}$	${J}_{3}$	${J}_{4}$	${J}_{5} \left(\times 1{0}^{-5}\right)$	${J}_{1}$	${J}_{2}$	${J}_{3}$	${J}_{4}$	${J}_{5} \left(\times 1{0}^{-5}\right)$
El Centro	CryStAl	0.264	0.285	0.152	0.157	1.780	0.284	0.235	0.258	0.248	0.550
	SPO	0.326	0.326	0.183	0.189	1.410	0.389	0.343	0.360	0.357	0.449
	PSO	0.313	0.319	0.176	0.182	1.490	0.384	0.231	0.344	0.243	0.551
	HS	0.326	0.327	0.182	0.188	1.420	0.521	0.312	0.427	0.329	0.420
	KH	0.426	0.402	0.252	0.259	0.980	0.567	0.388	0.470	0.410	0.436
	ACO	0.452	0.435	0.279	0.286	0.890	0.562	0.284	0.456	0.302	0.477
	GA	0.303	0.313	0.172	0.178	1.530	0.480	0.326	0.399	0.342	0.453
	GWO	0.330	0.329	0.186	0.193	1.380	0.319	0.234	0.265	0.248	0.577
	HHO	0.296	0.308	0.169	0.175	1.570	0.428	0.214	0.348	0.227	0.597
	SSA	0.380	0.361	0.211	0.216	1.170	0.546	0.649	0.515	0.519	0.361
	HO	0.334	0.331	0.189	0.195	1.352	0.468	0.258	0.382	0.272	0.787
	DSA	0.302	0.312	0.171	0.177	1.536	0.311	0.229	0.255	0.245	0.863
Bam	CryStAl	0.390	0.427	0.298	0.349	1.550	0.150	0.276	0.135	0.252	0.034
	SPO	0.475	0.461	0.366	0.417	1.210	0.351	0.545	0.289	0.479	0.027
	PSO	0.459	0.445	0.351	0.403	1.270	0.227	0.190	0.202	0.172	0.034
	HS	0.472	0.449	0.361	0.413	1.220	0.234	0.333	0.143	0.293	0.028
	KH	0.543	0.633	0.506	0.555	0.920	0.331	0.287	0.246	0.238	0.027
	ACO	0.605	0.710	0.555	0.602	0.890	0.275	0.168	0.165	0.146	0.029
	GA	0.451	0.448	0.345	0.399	1.310	0.223	0.377	0.151	0.320	0.028
	GWO	0.485	0.474	0.377	0.430	1.150	0.152	0.270	0.110	0.236	0.035
	HHO	0.442	0.449	0.340	0.394	1.350	0.152	0.241	0.097	0.206	0.036
	SSA	0.524	0.494	0.414	0.465	0.960	0.387	0.482	0.444	0.400	0.024
	HO	0.491	0.488	0.386	0.439	1.134	0.283	0.414	0.181	0.353	0.047
	DSA	0.447	0.443	0.342	0.394	1.133	0.161	0.273	0.114	0.227	0.052
Chi-Chi	CryStAl	0.286	0.277	0.195	0.198	1.530	0.238	0.239	0.210	0.252	0.169
	SPO	0.294	0.282	0.233	0.237	1.180	0.303	0.270	0.285	0.311	0.140
	PSO	0.294	0.283	0.224	0.228	1.260	0.303	0.400	0.298	0.425	0.169
	HS	0.296	0.284	0.231	0.234	1.210	0.357	0.360	0.279	0.472	0.147
	KH	0.346	0.375	0.319	0.325	0.890	0.371	0.440	0.335	0.477	0.145
	ACO	0.382	0.411	0.352	0.359	0.840	0.399	0.351	0.313	0.444	0.159
	GA	0.292	0.282	0.221	0.225	1.300	0.335	0.377	0.271	0.455	0.151
	GWO	0.293	0.281	0.238	0.243	1.170	0.262	0.251	0.231	0.313	0.178
	HHO	0.291	0.281	0.217	0.222	1.330	0.311	0.314	0.258	0.402	0.186
	SSA	0.297	0.288	0.263	0.267	1.000	0.351	0.422	0.343	0.575	0.120
	HO	0.291	0.281	0.243	0.249	1.154	0.319	0.392	0.257	0.464	0.259
	DSA	0.292	0.282	0.219	0.223	1.299	0.292	0.369	0.271	0.399	0.274
Turkey	CryStAl	0.279	0.340	0.209	0.232	0.760	0.168	0.265	0.096	0.247	0.734
	SPO	0.310	0.377	0.255	0.279	0.600	0.325	0.393	0.184	0.377	0.524
	PSO	0.304	0.370	0.244	0.269	0.620	0.239	0.206	0.135	0.192	0.736
	HS	0.310	0.377	0.252	0.276	0.590	0.275	0.409	0.175	0.386	0.529
	KH	0.442	0.448	0.362	0.388	0.440	0.304	0.353	0.200	0.313	0.508
	ACO	0.506	0.494	0.405	0.431	0.400	0.307	0.243	0.198	0.227	0.572
	GA	0.301	0.366	0.240	0.266	0.630	0.312	0.431	0.188	0.402	0.528
	GWO	0.314	0.381	0.262	0.288	0.590	0.201	0.256	0.110	0.236	0.766
	HHO	0.298	0.362	0.236	0.262	0.650	0.241	0.278	0.137	0.262	0.796
	SSA	0.347	0.407	0.290	0.315	0.510	0.314	0.503	0.212	0.504	0.414
	HO	0.323	0.383	0.268	0.294	0.586	0.286	0.378	0.180	0.348	0.937
	DSA	0.299	0.364	0.238	0.263	0.641	0.190	0.288	0.122	0.258	0.106
Kobe	CryStAl	0.275	0.241	0.195	0.194	1.600	0.229	0.250	0.198	0.246	1.680
	SPO	0.327	0.285	0.235	0.234	1.340	0.365	0.368	0.332	0.353	1.420
	PSO	0.316	0.275	0.226	0.225	1.380	0.274	0.292	0.235	0.286	1.682
	HS	0.328	0.286	0.233	0.233	1.330	0.451	0.399	0.425	0.424	1.551
	KH	0.441	0.397	0.323	0.325	1.150	0.462	0.554	0.470	0.567	1.502
	ACO	0.478	0.435	0.360	0.363	1.080	0.401	0.412	0.383	0.444	1.626
	GA	0.307	0.266	0.221	0.221	1.410	0.388	0.349	0.373	0.358	1.554
	GWO	0.332	0.289	0.239	0.240	1.370	0.270	0.253	0.251	0.249	1.770
	HHO	0.300	0.259	0.217	0.217	1.440	0.285	0.230	0.267	0.233	1.843
	SSA	0.378	0.334	0.268	0.268	1.240	0.413	0.653	0.435	0.601	1.229
	HO	0.336	0.293	0.243	0.244	1.380	0.401	0.273	0.389	0.287	1.637
	DSA	0.306	0.265	0.219	0.219	1.412	0.200	0.244	0.205	0.251	1.725
Northridge	CryStAl	0.577	0.608	0.411	0.425	1.480	0.211	0.287	0.159	0.283	0.975
	SPO	0.595	0.641	0.478	0.493	1.230	0.331	0.432	0.278	0.419	0.819
	PSO	0.596	0.625	0.464	0.478	1.280	0.264	0.280	0.199	0.277	0.975
	HS	0.598	0.635	0.475	0.489	1.230	0.365	0.378	0.314	0.379	0.858
	KH	0.670	0.762	0.629	0.646	0.901	0.425	0.482	0.416	0.464	0.857
	ACO	0.704	0.796	0.692	0.708	0.831	0.373	0.346	0.322	0.345	0.913
	GA	0.595	0.621	0.457	0.472	1.320	0.362	0.388	0.319	0.390	0.870
	GWO	0.598	0.656	0.488	0.504	1.230	0.242	0.267	0.200	0.279	1.023
	HHO	0.593	0.618	0.450	0.466	1.360	0.252	0.252	0.206	0.255	1.053
	SSA	0.602	0.686	0.530	0.543	1.020	0.418	0.263	0.414	0.244	0.731
	HO	0.596	0.665	0.496	0.513	1.227	0.379	0.325	0.338	0.321	1.490
	DSA	0.593	0.616	0.453	0.469	1.321	0.245	0.268	0.234	0.257	1.573

Considerable variations can be seen in the performance of the algorithms across different earthquakes in both examples. In the 3-story structure, for the El Centro earthquake, the CryStAl algorithm demonstrates its superiority with the lowest ${J}_{1}$ value of 0.264, outperforming SPO (${J}_{1}=$ 0.326), PSO (${J}_{1}=$ 0.313), HS (${J}_{1}=$ 0.326), KH (${J}_{1}=$ 0.426), ACO (${J}_{1}=$ 0.452), GA (${J}_{1}=$ 0.303), GWO (${J}_{1}=$ 0.330), HHO (${J}_{1}=$ 0.296), SSA (${J}_{1}=$ 0.380), HO (${J}_{1}=$ 0.334), and DSA (${J}_{1}=$ 0.302). In terms of minimizing displacement, CryStAl performs approximately 23.7%, 15.7%, 23.4%, 61.8%, 71.7%, 43.2%, 10.5%, 10.4%, 30.5%, 21.0%, and 12.6% better than SPO, PSO, HS, KH, ACO, GA, GWO, HHO, SSA, HO, and DSA, respectively. For index ${J}_{2}$, CryStAl (${J}_{2}$= 0.285) once again demonstrates its efficiency, outperforming SPO (${J}_{2}$= 0.326), PSO (${J}_{2}$= 0.319), HS (${J}_{2}$= 0.327), KH (${J}_{2}$= 0.402), ACO (${J}_{2}$= 0.435), GA (${J}_{2}$=0.313), GWO (${J}_{2}$=0.329), HHO (${J}_{2}$=0.308), SSA (${J}_{2}$=0.361), HO (${J}_{2}$=0.331), and DSA (${J}_{2}$=0.312). In terms of reducing acceleration, the performance of CryStAl is around 12.1, 10.4, 12.9, 29.2, 34.6, 8.95, 13.37, 7.47, 21.05, 16.1, and 9.5% better than SPO, PSO, HS, KH, ACO, GA, GWO, HHO, SSA, HO, and DSA, respectively.

Considering indices ${J}_{3}$ and ${J}_{4}$, CryStAl once again emerges as the top performer with ${J}_{3}$= 0.152 and ${J}_{4}$= 0.157, outperforming the other algorithms in minimizing both displacement and acceleration. On the other hand, in terms of index ${J}_{5}$, CryStAl turns out to be the poorest algorithm, and though the differences between the algorithms are relatively small in terms of this index, CryStAl demonstrates a relatively high control force. The values of index ${J}_{5}$ in the CryStAl algorithm for the 3-story building are respectively 24.78, 26.42, 24.94, 17.43, 20.45, and 25.16% higher than the average of that index of the other algorithms in the El Centro, Bam, Chi-Chi, Kobe, Northridge, and Turkey earthquakes, respectively. This indicates that the CryStAl algorithm yields greater force and superior results in all other indices for the 3-story building. The comparison in the 6-story example is more favorable for CryStAl. In some earthquake scenarios, such as Chi-Chi and Northridge, CryStAl even outperforms the average performance of other algorithms. This suggests that CryStAl requires lower forces to achieve the desired outcome compared to other algorithms.

In addition to comparing CryStAl with the other algorithms, it is important to note that the performance trends of different algorithms can vary across various indices. For instance, under the El Centro earthquake, the HHO algorithm achieves a higher score (0.296) in ${J}_{1}$, while the GA algorithm performs slightly worse (0.303). However, examining the same case for the 6-story structure, we observe that the difference between these two algorithms under this criterion is greater. In terms of index ${J}_{2}$, the SPO algorithm obtains almost the same score as the HS algorithm.

Similar to the 3-story building, in the case of the 6-story one, the CryStAl algorithm demonstrates its superiority over other algorithms in almost all the considered earthquakes. For instance, in terms of index ${J}_{1}$, the CryStAl algorithm performs 37.44% better than the average of all algorithms under all examined earthquakes. However, it is crucial not to overlook the excellent performance of the GWO and DSA algorithms, which managed to achieve a similar level of performance as CryStAl in all earthquakes and outperform the other well-established algorithms. Furthermore, other algorithms such as HHO, SPO, and HO show commendable performance in most cases. As for the worst-performing algorithm, KH may be mentioned, which can be deemed the worst algorithm in half of the earthquakes in terms of index ${J}_{1}$. However, in earthquakes like the one in Turkey, it performed better than algorithms such as GA, SSA, and SPO.

The DSA algorithm shows promise, particularly for the 3-story building. For the El Centro earthquake, DSA achieves indices between 0.302 (3-story) and 0.311 (6-story) for top-story displacement, often competing with the corresponding CryStAl values of 0.264 and 0.284. This trend continues across other earthquakes and other performance indices, suggesting DSA’s effectiveness, especially for smaller structures. Similar to DSA, HO demonstrates competitive performance across different earthquakes, turning out to be particularly effective for the 3-story building in some cases. For instance, for the El Centro earthquake, HO's top-story displacement index ranges from 0.296 (3-story) to 0.491 (6-story), which is comparable to CryStAl’s results.

**Figure 5:**
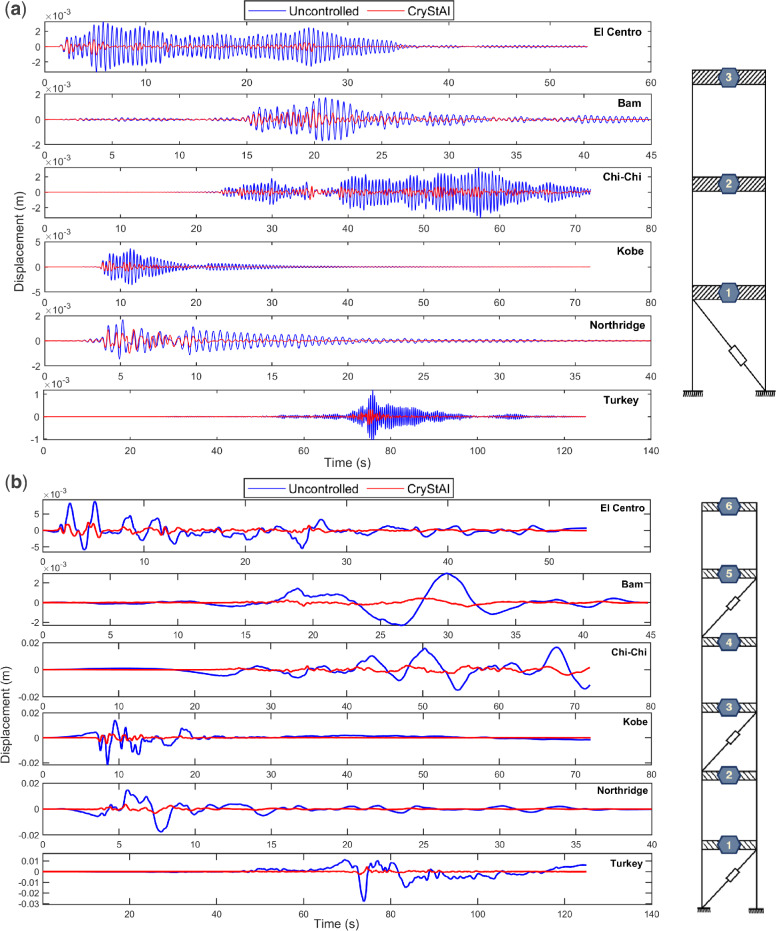
Displacement response of structures under different earthquakes with and without using the CryStAl algorithm. (**a**) The 3-story building. (**b**) The 6-story building.

**Figure 6:**
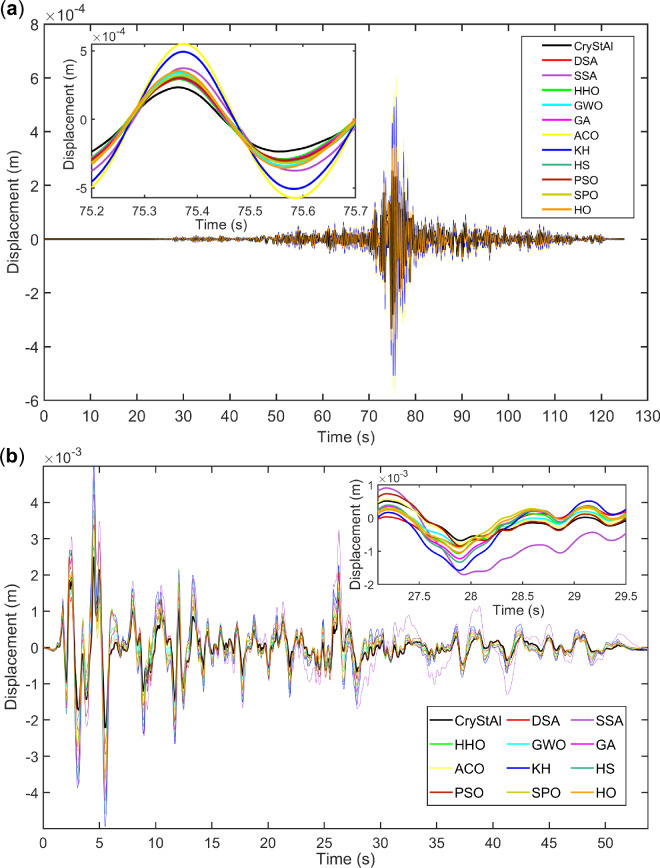
Comparison of the performances of various optimization algorithms. (**a**) Displacement response of the 3-story structure under the Turkey earthquake. (**b**) Displacement response of the 6-story structure under the El-Centro earthquake.

For further investigation, Fig. 5 shows the displacement responses of the structures during the aforementioned earthquakes. Figure 5a,b show the controlled responses achieved by the FOPID controller optimized using the CryStAl algorithm compared to those of the uncontrolled structure. The performance of the CryStAl algorithm in mitigating the seismic response through FOPID controller optimization has been evaluated. Figure 6a,b show displacement responses of the 3-story and 6-story structures under different earthquakes and CryStAl algorithms, respectively. Figure 5 provides additional evidence of the CryStAl algorithm's effectiveness in significantly reducing structural response compared to other algorithms.

Figure 6 provides a comparison of the responses of various algorithms to showcase the differences between these algorithms, underscoring the high performance of the CryStAl algorithm in markedly diminishing structural vibrations. It is important to note that the results of other algorithms are qualitatively similar, making it difficult to discern the differences at this scale; to address this issue, a zoomed-in view of a part of the chart is provided in these figures to facilitate easier comparison between the algorithms. Figure 6a,b present the displacement responses of controlled structures under the Turkey and El-Centro earthquakes, respectively, using all the algorithms explored in this paper. In both cases, CryStAl demonstrated good performance in reducing seismic response.

Overall, the algorithms exhibited qualitatively similar behaviors in the two examples examined in this paper. However, the value of structural response reduction depends on various factors, including the properties of the structure, the earthquake load, and the controller. While structural properties and earthquake load characteristics remain constant during the optimization process iterations, the only factor that changes is the controller’s performance which is based on the inherent behavior of metaheuristic algorithms. Therefore, this process is repeated numerous times to obtain a relatively consistent pattern in controller behavior, allowing for a comparison of these controllers.

Table 8 presents a comparison of different algorithms used for earthquake analysis based on the reduction percentage and the absolute maximum displacement, velocity, and acceleration achieved. As can be seen from this table, during the El Centro earthquake, the Crystal algorithm demonstrated the most significant reduction, with percentages of 73.60 and 71.57%, corresponding to absolute maximum displacements of 0.88 and 2.50 mm, respectively, for the 3-story and 6-story structures. While ACO, PSO, HS, KH, SPO, GA, GWO, HHO, SSA, HO, and DSA achieved lower decrease percentages.

**Table 8:** Results of different algorithms in reducing displacement/velocity/acceleration for various earthquakes.

Earthquake	Algorithm	3-story structure						6-story structure					
		Displacement		Velocity		Acceleration		Displacement		Velocity		Acceleration	
		Decrease (%)	Absolute maximum (mm)	Decrease (%)	Absolute maximum (mm/s)	Decrease (%)	Absolute maximum ($\text{mm}/{\text{s}}^{2}$)	Decrease (%)	Absolute maximum (mm)	Decrease (%)	Absolute maximum (mm/s)	Decrease (%)	Absolute maximum ($\text{mm}/{\text{s}}^{2}$)
El Centro	CryStAl	73.60	0.88	67.60	14.36	71.52	192.69	71.57	2.50	67.92	10.12	75.65	166.36
	SPO	54.76	1.51	54.52	20.16	56.49	294.33	61.08	3.43	60.27	12.54	64.37	243.38
	PSO	68.73	1.04	64.59	15.69	68.11	215.71	61.56	3.38	67.96	10.11	76.04	163.66
	HS	67.43	1.09	63.86	16.02	67.29	221.29	47.89	4.59	51.84	15.20	67.60	221.37
	KH	57.39	1.42	50.42	7.94	55.22	106.71	43.25	5.00	54.69	14.30	59.75	274.97
	ACO	67.37	1.09	63.94	15.98	67.42	220.37	43.76	4.95	55.94	13.91	70.49	201.59
	GA	69.66	1.01	65.12	15.46	68.68	211.87	52.05	4.22	55.18	14.15	66.18	231.07
	GWO	66.97	1.10	63.71	16.08	67.14	222.32	68.07	2.81	67.64	10.21	75.73	165.81
	HHO	70.44	0.99	65.59	15.25	69.21	208.31	57.20	3.77	67.49	10.26	77.77	151.85
	SSA	62.04	1.27	61.06	17.26	63.92	244.11	45.42	4.81	54.61	14.32	42.30	394.18
	HO	65.77	1.12	63.65	16.11	66.41	223.85	57.86	3.71	48.19	16.35	69.27	209.93
	DSA	69.77	1.00	65.22	15.41	68.85	210.75	68.89	2.73	70.34	9.36	76.22	162.45
Bam	CryStAl	61.04	0.68	56.90	10.92	57.32	155.60	85.04	0.44	66.79	0.96	67.86	28.58
	SPO	39.53	1.06	30.51	17.60	29.05	258.62	64.86	1.03	54.34	1.32	36.58	56.39
	PSO	54.13	0.80	49.23	12.86	55.47	162.32	77.33	0.67	71.95	0.81	77.85	19.70
	HS	52.81	0.83	48.16	13.13	55.10	163.68	76.57	0.69	32.48	1.95	61.30	34.41
	KH	45.69	0.95	37.96	15.71	36.75	230.57	66.93	0.97	57.90	1.22	66.66	29.65
	ACO	52.53	0.83	47.26	13.36	53.92	167.98	72.53	0.81	75.31	0.71	80.47	17.36
	GA	54.93	0.79	49.55	12.78	55.20	163.31	77.68	0.65	42.02	1.68	56.16	38.98
	GWO	51.54	0.85	45.68	13.76	52.57	172.90	84.79	0.45	56.92	1.25	68.57	27.95
	HHO	55.76	0.78	50.02	12.66	55.12	163.60	84.82	0.45	51.12	1.41	71.99	24.90
	SSA	47.65	0.92	43.01	14.43	50.56	180.22	61.31	1.14	56.48	1.26	43.94	49.85
	HO	50.87	0.86	44.38	14.09	51.05	178.15	71.71	0.83	36.59	1.83	51.59	18.34
	DSA	55.32	0.78	50.13	12.62	55.69	161.54	83.85	0.47	49.94	1.45	68.20	28.27
Chi-Chi	CryStAl	71.40	0.95	76.49	11.21	72.26	177.68	76.23	3.94	85.92	2.13	76.08	229.40
	SPO	61.86	1.27	60.50	18.83	58.91	263.19	69.66	5.03	75.63	3.69	72.99	259.06
	PSO	70.57	0.98	74.08	12.36	71.69	181.35	69.67	5.03	55.39	6.76	59.99	383.66
	HS	70.37	0.99	73.69	12.55	71.58	182.04	64.31	5.91	75.76	3.67	63.99	345.33
	KH	65.43	1.15	64.05	17.14	62.52	240.05	62.86	6.16	66.24	5.12	55.98	422.16
	ACO	70.64	0.98	73.64	12.57	71.80	180.63	60.05	6.62	57.79	6.40	64.92	336.38
	GA	70.76	0.97	74.29	12.26	71.80	180.66	66.49	5.55	79.67	3.08	62.27	361.86
	GWO	70.67	0.98	72.78	12.98	71.88	180.11	73.76	4.35	76.85	3.51	74.91	240.62
	HHO	70.93	0.97	74.52	12.15	71.87	180.17	68.87	5.16	79.02	3.18	65.50	330.89
	SSA	70.32	0.99	72.06	13.32	71.16	184.74	64.87	5.82	75.76	3.67	57.80	404.72
	HO	69.68	0.96	71.69	13.50	71.85	180.28	68.08	5.28	70.62	4.45	60.78	37,610
	DSA	70.79	0.97	74.45	12.18	71.80	180.63	70.82	4.83	64.47	5.38	63.14	353.48
Kobe	CryStAl	72.47	1.02	74.93	12.53	75.94	177.13	64.63	4.98	68.18	29.64	68.42	156.20
	SPO	52.24	1.76	56.76	21.60	56.54	319.93	43.67	7.93	59.21	37.99	53.65	229.23
	PSO	68.41	1.17	71.82	14.08	72.54	202.10	57.76	5.95	63.66	33.85	73.14	132.87
	HS	67.25	1.21	71.20	14.39	71.43	210.34	30.55	9.78	36.24	59.39	49.70	248.78
	KH	55.95	1.63	60.84	19.56	60.26	292.53	28.82	10.02	30.99	64.28	30.10	345.72
	ACO	67.27	1.21	71.09	14.44	71.49	209.84	38.12	8.71	32.79	62.61	48.00	257.18
	GA	69.25	1.14	72.18	13.90	73.40	195.85	40.26	8.41	49.97	46.60	55.98	217.70
	GWO	66.79	1.23	70.72	14.62	71.05	213.09	58.44	5.85	66.60	31.11	68.04	158.06
	HHO	70.01	1.11	72.53	13.72	74.14	190.34	56.10	6.18	65.92	31.75	71.02	143.34
	SSA	62.21	1.40	67.75	16.11	66.60	245.87	36.35	8.96	43.27	52.85	39.86	297.46
	HO	66.45	1.23	70.31	14.83	68.69	215.95	38.17	8.70	49.95	46.63	65.60	170.12
	DSA	69.39	1.13	72.34	13.81	73.49	195.08	69.15	4.32	65.81	31.85	69.25	152.09
Northridge	CryStAl	42.30	0.97	43.80	12.98	39.18	190.32	74.83	3.72	69.45	23.41	66.17	244.38
	SPO	29.61	1.18	17.73	19.00	20.36	249.21	60.47	5.83	62.42	28.80	49.01	368.34
	PSO	40.42	1.00	39.84	13.90	37.48	195.64	68.46	4.65	69.47	23.40	66.98	238.54
	HS	40.22	1.00	38.31	14.25	36.46	198.82	56.34	6.44	54.62	34.78	55.41	322.11
	KH	32.96	1.13	21.65	18.10	23.76	238.57	49.22	7.49	58.32	31.95	43.12	410.85
	ACO	40.46	1.00	37.52	14.43	35.86	200.71	55.45	6.57	58.48	31.82	59.19	294.80
	GA	40.52	1.00	40.61	13.72	37.86	194.46	56.77	6.38	58.77	31.60	54.17	331.02
	GWO	40.21	1.00	35.89	14.81	34.44	205.15	71.06	4.27	70.38	22.71	68.50	227.55
	HHO	40.66	1.00	41.47	13.52	38.16	193.50	69.93	4.44	68.87	23.86	70.32	214.37
	SSA	39.76	1.01	31.72	15.77	31.36	214.79	50.05	7.37	57.09	32.89	68.95	224.30
	HO	40.38	1.00	34.48	15.14	28.55	208.18	54.66	6.69	59.87	30.75	61.70	276.61
	DSA	40.69	0.99	41.15	13.59	38.37	192.87	70.69	4.32	67.73	24.74	68.37	228.44
Turkey	CryStAl	72.10	0.33	69.70	4.85	66.00	81.02	57.95	4.68	72.34	17.83	71.74	304.87
	SPO	49.43	0.61	45.75	8.69	50.60	117.72	18.75	9.04	67.29	21.08	53.79	498.53
	PSO	69.60	0.36	66.19	5.41	62.98	88.22	40.25	6.65	72.29	17.86	77.99	237.52
	HS	69.04	0.37	65.16	5.58	62.31	89.81	31.22	7.65	54.11	29.58	56.27	471.80
	KH	55.79	0.53	50.42	7.94	55.22	106.71	23.99	8.45	54.47	29.35	59.19	440.29
	ACO	69.03	0.37	64.72	5.65	62.32	89.80	23.10	8.55	56.90	27.78	74.00	280.49
	GA	69.93	0.36	66.75	5.32	63.39	87.25	21.94	8.68	56.75	27.88	53.96	496.78
	GWO	68.62	0.38	63.78	5.80	61.85	90.90	49.76	5.59	68.20	20.50	72.68	294.79
	HHO	70.25	0.36	66.95	5.29	63.80	86.26	39.69	6.71	66.96	21.30	70.33	320.11
	SSA	65.34	0.41	60.27	6.36	59.33	96.91	21.36	8.75	58.94	26.47	46.25	579.94
	HO	67.74	0.38	62.58	5.99	55.16	91.18	28.29	7.97	59.95	25.81	59.61	435.79
	DSA	70.07	0.35	66.97	5.29	63.59	86.77	52.34	5.30	63.56	23.49	69.18	332.49

In the case of the 3-story building, in terms of displacement reduction during the El Centro earthquake, the algorithms closest in performance to CryStAl are the HHO and DSA, with reduction percentages of 70.44 and 69.77%, respectively. Similarly, in the case of the 6-story structure, the DSA algorithm exhibited the closest performance with a reduction percentage of 68.89%. Interestingly, the HHO algorithm even outperformed CryStAl in reducing the structural acceleration response by 2.12%. Comparable performance was also observed for this algorithm for the Bam, Kobe, and Northridge earthquakes. Under the Bam earthquake, HHO exhibited the closest performance to CryStAl in terms of displacement response reduction in the 3-story example. In terms of velocity and acceleration response reduction, the HHO algorithm had the second-highest reduction after CryStAl in the 3-story example. However, in the 6-story example, CryStAl ranked third. after PSO and DSA, in terms of velocity response reduction. Moreover, HHO again had the best performance in terms of acceleration response reduction in this example.

On average, the CryStAl, SPO, PSO, HS, KH, ACO, GA, GWO, HHO, SSA, HO, and DSA algorithms achieved displacement reductions of 65.48, 47.91, 61.98, 52.20, 61.21, 62.51, 60.80, 63.00%, 57.89, 60.15, and 62.67%, respectively, across all earthquakes in the case of the 3-story structure. For the 6-story structure, while this trend is qualitatively similar, the optimized controllers generally exhibited greater reductions in acceleration compared to the 3-story structure. In the 3-story example, the HHO algorithm achieved an acceleration response reduction 2.31% lower than that of CryStAl. From this comparison, it can be concluded that CryStAl performed better than HHO and the others in reducing displacement for the El Centro and almost all other earthquakes. In the Bam earthquake, CryStAl realized a decrease of 61.04% in the case of the 3-story structure for displacement response, while SPO, ACO, PSO, HS, KH, GA, GWO, HHO, SSA, HO, and DSA achieved lower decreases.

For the Chi-Chi earthquake, in the case of the 3-story structure, the CryStAl and HHO algorithms demonstrated displacement reductions of 71.40 and 70.93%, respectively. These algorithms achieved reductions of 76.23 and 68.87% in the case of the 6-story structure for the same earthquake. This comparison between the CryStAl and HHO algorithms illustrates their nearly similar behavior across different earthquake scenarios and various structures. In the case of the Kobe earthquake, for the 3-story example, the Crystal algorithm achieved a decrease of 72.47%, while the SPO algorithm (52.24%) turned out to be worse than HHO (70.01%), GWO (66.79%), SSA (62.21%), and DSA (69.36%). In the 6-story example, the DSA and GWO algorithms outperformed the HHO algorithm.

In the 3-story structure, the CryStAl algorithm achieved decreases of 42.30 and 72.10% for the Northridge and Turkey earthquakes, respectively. Overall, the CryStAl algorithm consistently achieves high decrease percentages and shows promising performance in reducing earthquake displacement. For the El Centro earthquake, the CryStAl, PSO, SPO, HS, KH, ACO, GA, GWO, HHO, SSA, HO, and DSA algorithms demonstrate significant reductions of 67.60, 54.52, 64.59, 63.86, 50.42, 63.936, 65.12, 63.71, 65.59, 61.06, 63.65, and 65.22% in the absolute maximum velocity, respectively; for the Bam earthquake, CryStAl again exhibits the highest reduction percentage of 56.90%, while SPO displays the lowest reduction percentage of 30.51%. The results of the remaining algorithms fall between these two extremes, with HHO at 50.02%, GA at 49.55%, PSO at 49.23%, HSM at 48.16%, GWO at 4568%, SSA at 43.01,% KH at 36.75%, HO at 44.38%, and DSA at 50.13%. In the case of the Chi-Chi earthquake, for the 3-story example, the reduction percentage varies between 60.50 and 76.49%, obtained by the SPO and CryStAl algorithms producing the highest and lowest values, respectively. Similarly, for the other earthquakes, the CryStAl algorithm achieved relatively high reduction percentages, indicating promising performance in mitigating earthquake-induced velocities.

The CryStAl algorithm consistently stands out by achieving significant decreases in acceleration across all earthquakes. It demonstrates an average reduction of 63.73 (3-story) and 70.98% (6-story), varying between 20.36 and 75.94% (3-story), and 30.10% and 80.47%(6-story), indicating its effectiveness for this seismic application. The HHO and DSA algorithms also deliver significant decreases in acceleration, demonstrating their potential for earthquake control.

**Figure 7:**
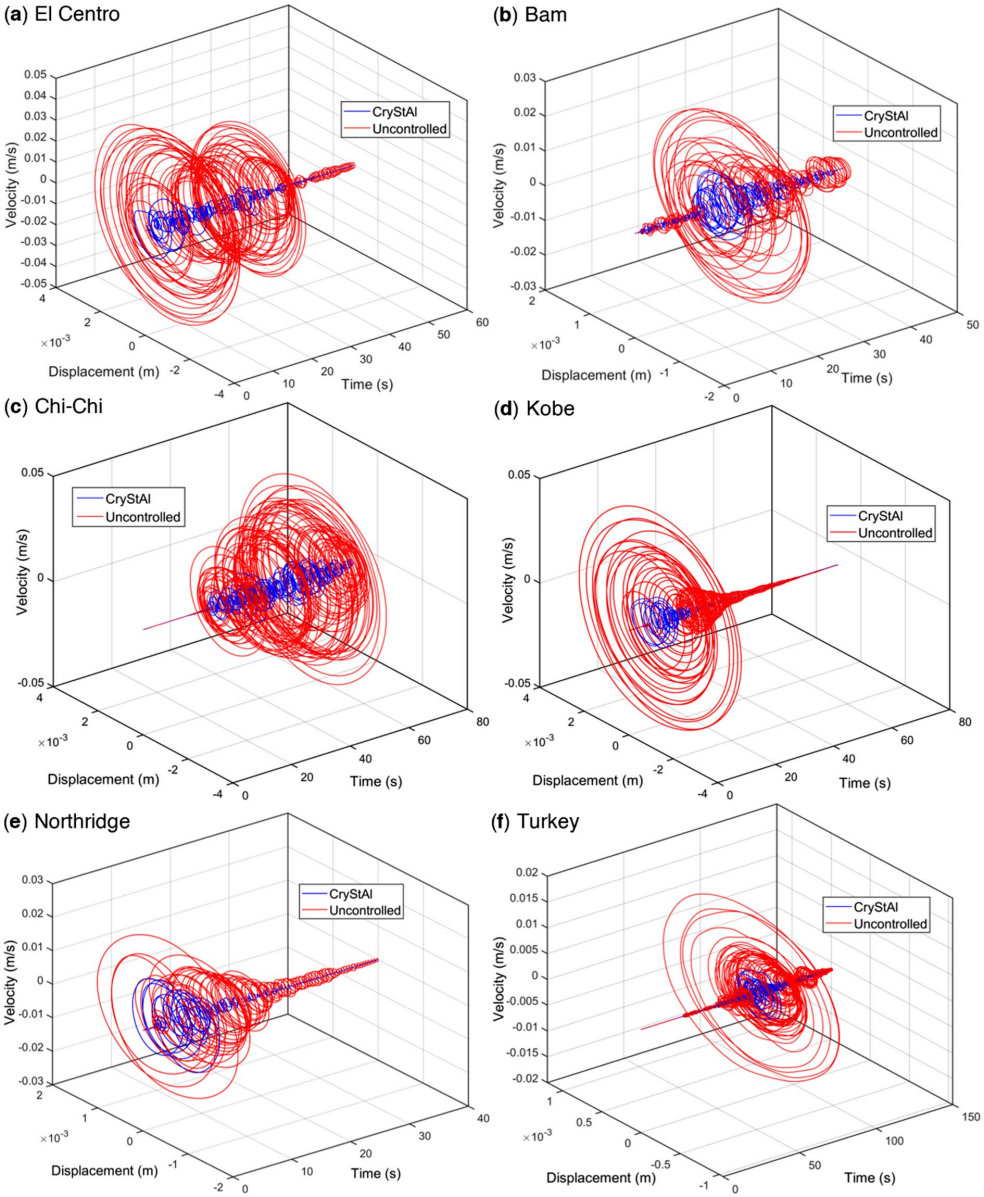
3D plots of the uncontrolled and FOPID-controlled 3-story structures under the various earthquakes.

Figure 7 illustrates the displacement and velocity of the third floor over time in both the uncontrolled and FOPID-controlled 3-story structures. This figure specifically displays the velocity responses of CryStAl under all the earthquakes. According to this plot, the controllers developed based on CryStAl are capable of effectively diminishing the displacement and velocity responses simultaneously.

**Figure 8:**
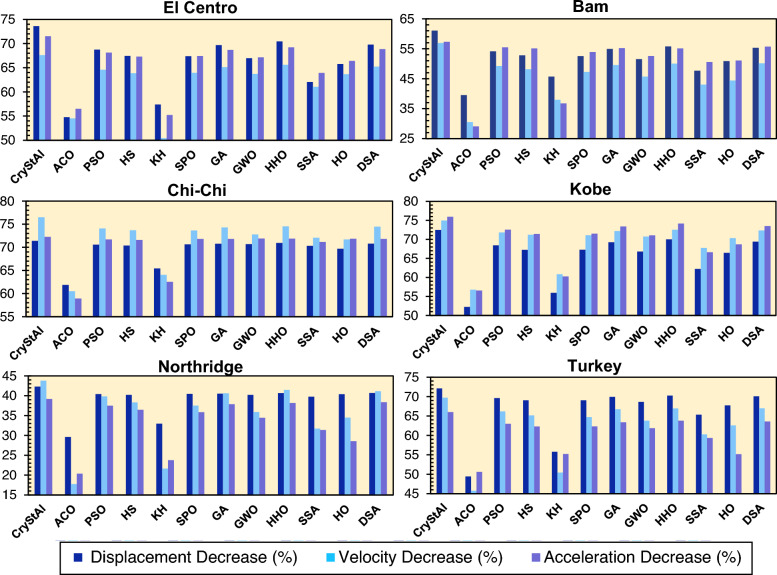
Performance of various algorithms in terms of displacement, velocity, and acceleration reductions for the 3-story structure under each earthquake scenario.

Figures 8. 9 depict the displacement, velocity, and acceleration reductions achieved by all algorithms, demonstrating their effectiveness in controlling structural responses across the various earthquake scenarios.

**Figure 9:**
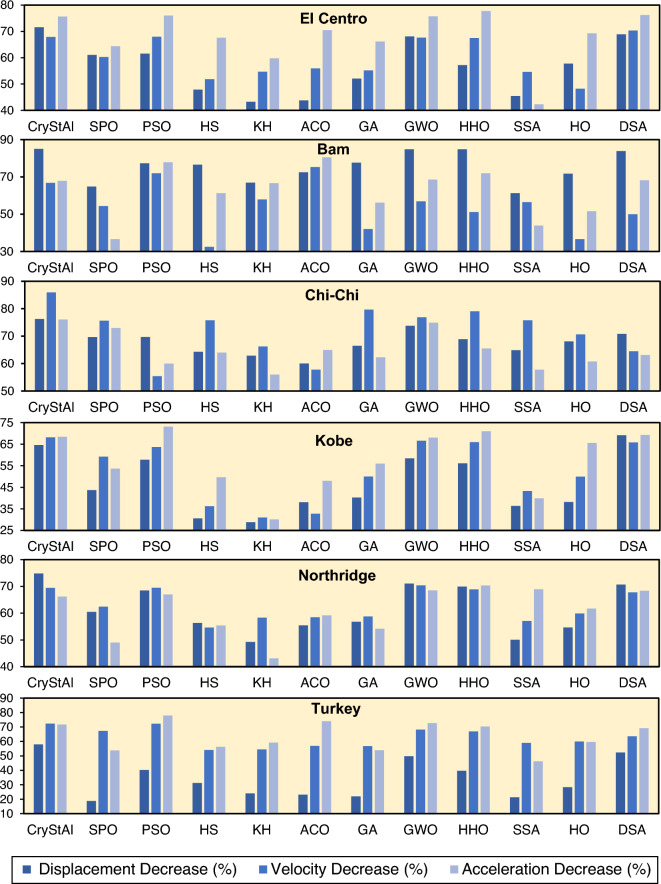
Performance of various algorithms in terms of displacement, velocity, and acceleration reductions for the 6-story structure under each earthquake scenario.

According to Figs. 8. 9, by comparing the performance of the different algorithms, it can be concluded that CryStAl consistently outperforms other algorithms in terms of reducing the displacement, velocity, and acceleration responses under all earthquakes. Despite the fact that, in some cases, other algorithms such as DSA and HHO, manage to achieve better performance in reducing responses, CryStAl achieves the highest percentage decrease in these values, indicating its effectiveness in optimizing the FOPID controller.

For the 3-story structure, Crystal reduces the displacement, velocity, and acceleration responses by 65.49, 64.90, and 63.70%, respectively, which is higher compared to the average reduction of the other algorithms by 59.23, 57.28, and 58.35%, respectively. This means that CryStAl is more successful in reducing acceleration. Moreover, in the 3-story example, HHO, SPO, GWO, and DSA turn out to be reliable algorithms, leading to considerable improvements in structural responses. For the 6-story structure, CryStAl provides exceptional reductions of 71.71% for displacement, 71.77% for velocity, and 70.98% for acceleration. Notably, these gains significantly exceed the average performance of other algorithms, which are 55.75, 59.79, and 62.34%, respectively, for the same measures.

Table 9 summarizes the control force of a FOPID controller, optimized by various algorithms for different earthquakes. The table provides the RMS values and the absolute maximum values of the control force during each earthquake for the two structures. According to Table 9, in the 3-story structure, SSA exhibited lower RMS force than other algorithms in two out of six earthquakes, ACO showed lower RMS force in three earthquakes, and DSA had the lowest RMS force in one case, i.e., under the Chi-Chi earthquake. Despite having lower RMS force values, the SSA, ACO, and DSA algorithms still demonstrated satisfactory performance. Notably, some algorithms, such as CryStAl, achieved a higher reduction in RMS force, but they also applied a higher control force. In the 6-story structure, the SSA algorithm recorded the lowest force; however, the results obtained from this lower force were not desirable in terms of other metrics.

**Table 9:** Comparison of RMS and absolute maximum force for different earthquakes and algorithms.

Earthquake	Algorithm	3-story structure		6-story structure	
		RMS of force (KN)	Absolute maximum of force (KN)	RMS of force (KN)	Absolute maximum of force (KN)
El Centro	CryStAl	1.9866	0.0535	6.3628	0.1997
	PSO	1.7759	0.0446	5.4662	0.1628
	SPO	1.7196	0.0423	6.3683	0.1999
	HS	1.7314	0.0426	6.2398	0.1526
	KH	1.3671	0.0295	6.0228	0.1582
	ACO	1.2875	0.0270	6.3863	0.1732
	GA	1.8062	0.0458	6.1240	0.1643
	GWO	1.1437	0.0414	6.7043	0.2093
	HHO	1.2441	0.0469	6.9552	0.2165
	SSA	1.0270	0.0352	5.0368	0.1309
	HO	1.6854	0.0405	6.281	0.1736
	DSA	1.2275	0.0461	6.376	0.1902
Bam	CryStAl	1.7849	0.0465	0.4910	0.0124
	PSO	1.5476	0.0382	0.3857	0.0097
	SPO	1.4952	0.0364	0.4916	0.0124
	HS	1.4944	0.0365	0.3853	0.0103
	KH	1.0901	0.0277	0.3891	0.0098
	ACO	1.0011	0.0266	0.4138	0.0107
	GA	1.5857	0.0392	0.3962	0.0101
	GWO	1.1278	0.0346	0.5105	0.0127
	HHO	1.1468	0.0406	0.5241	0.0131
	SSA	1.1068	0.0288	0.3431	0.0086
	HO	1.4633	0.03402	0.4134	0.0103
	DSA	1.1451	0.04000	0.4506	0.0114
Chi-Chi	CryStAl	2.6284	0.0460	3.0868	0.0613
	PSO	2.3458	0.0379	2.7565	0.0509
	SPO	2.2727	0.0355	3.0886	0.0614
	HS	2.2746	0.0364	3.1801	0.0532
	KH	1.7987	0.0270	3.0776	0.0528
	ACO	1.6927	0.0251	3.2101	0.0577
	GA	2.4025	0.0390	3.0998	0.0548
	GWO	1.2057	0.0351	3.2589	0.0648
	HHO	1.3369	0.0399	3.3697	0.0676
	SSA	1.0416	0.0301	4.0436	2.7095
	HO	2.255	0.03464	3.1591	0.0572
	DSA	1.013	0.03899	3.1703	0.0603
Kobe	CryStAl	1.7622	0.0479	16.3817	0.6097
	PSO	1.5822	0.0413	14.1179	0.5154
	SPO	1.5324	0.0403	16.3955	0.6103
	HS	1.5400	0.0399	16.1476	0.5629
	KH	1.2286	0.0344	15.4884	0.5452
	ACO	1.1673	0.0325	16.5284	0.5899
	GA	1.6140	0.0423	15.8294	0.5638
	GWO	1.6291	0.0411	17.2638	0.6424
	HHO	1.1009	0.0431	17.9426	0.6686
	SSA	0.9340	0.0371	12.8768	0.4459
	HO	1.5108	0.0414	16.2250	0.5814
	DSA	1.0001	0.0423	16.4154	0.6009
Northridge	CryStAl	1.5276	0.0443	10.1296	0.3536
	PSO	1.3232	0.0384	8.4349	0.2973
	SPO	1.2719	0.0369	10.1396	0.3536
	HS	1.2770	0.0368	9.3898	0.3112
	KH	0.9603	0.0270	9.1159	0.3108
	ACO	0.8984	0.0249	9.6992	0.3314
	GA	1.3576	0.0397	9.2685	0.3156
	GWO	1.0236	0.0369	10.6303	0.3712
	HHO	1.0289	0.0406	10.9986	0.3821
	SSA	1.0185	0.0305	7.6351	0.2652
	HO	1.2504	0.0367	9.5590	0.3287
	DSA	1.0287	0.0396	9.8770	0.3469
Turkey	CryStAl	0.6773	0.0230	7.4880	0.2664
	PSO	0.5923	0.0186	6.4003	0.1902
	SPO	0.5735	0.0180	7.4945	0.2671
	HS	0.5732	0.0180	7.3424	0.1919
	KH	0.4417	0.0133	7.1234	0.1843
	ACO	0.4167	0.0121	7.4738	0.2077
	GA	0.6059	0.0190	7.1793	0.1916
	GWO	0.5951	0.0176	7.8842	0.2781
	HHO	0.8387	0.0195	8.1509	0.2888
	SSA	0.6333	0.0153	6.0394	0.1501
	HO	0.5644	0.0176	7.3603	0.2068
	DSA	0.8612	0.0192	7.4927	0.2346

In general, a relatively high force could lead to undesirable consequences, such as structural damage or excessive vibration. Therefore, it is essential to carefully weigh the benefits and drawbacks of these algorithms before selecting them for a specific application. On the other hand, upon closer examination, it becomes evident that lower forces come at a cost. Overall, the findings of this analysis emphasize the importance of thoroughly evaluating and selecting the appropriate algorithm for each specific structure and seismic scenario.

### Statistical analysis of structural response reduction

This section investigates the effectiveness of various structural analysis algorithms in mitigating vibrations in the two building models using a non-parametric statistical test, namely, the Friedman test. The primary objective is to identify statistically significant differences in their ability to reduce displacement, velocity, and acceleration responses compared to the baseline algorithm CryStAl. The analysis reveals that CryStAl generally outperforms other algorithms across both building models and response metrics. For displacement, while CryStAl exhibits higher values than some algorithms like SPO, HS, and KH, it consistently shows notable reductions in velocity and acceleration compared to most, including SPO, PSO, KH, HHO, and SSA. These differences are deemed statistically significant, indicating CryStAl’s overall effectiveness in vibration control. However, it is important to note that statistical significance is not the only criterion; evaluating the magnitude of the difference between algorithms is also important.

Table 10 presents the results of the Friedman test across three response metrics (i.e., displacement, velocity, and acceleration) for both 3-story and 6-story building models. It reveals statistically significant differences (*p*-value < 0.05) in all cases, indicating that at least one algorithm (i.e., CryStAl) performs distinctly in reducing vibrations compared to others.

**Table 10:** Results of the Friedman test.

Response metric	3-story		6-story	
	Chi-square	*p*-value	Chi-square	*p*-value
Displacement	70.527	< 0.05	65.098	< 0.05
Velocity	71.407	< 0.05	50.609	< 0.05
Acceleration	65.802	< 0.05	53.318	< 0.05

Finally, this statistical analysis provides valuable insights into the effectiveness of different algorithms for vibration suppression. While CryStAl emerges as a promising candidate, careful consideration of its limitations is crucial for drawing definitive conclusions and translating these findings into practical applications.

## Conclusions

This study investigated the performance of two shear model structures, one with 3-stories and the other one with 6-stories, equipped with an active tendon system under various earthquake excitations. The analyses were based on the given properties of the structure, as well as the data from the El Centro, Kobe, Northridge, Chi-Chi, Bam, and Turkey earthquakes. Five performance indices were used to evaluate the simulation results, including the peak top-story displacement, peak top-story acceleration, RMS top-story displacement, RMS top-story acceleration, and control force. Twelve different optimization algorithms, namely CryStAl, SPO, PSO, HS, KH, ACO, GA, GWO, HHO, SSA, HO, and DSA, were used to solve the problem, followed by analyzing and comparing their results.

The CryStAl algorithm demonstrated significant advantages over several algorithms, including HHO and DSA, in reducing structural responses induced by the El Centro earthquake. For both buildings, CryStAl achieved the highest percentages of decrease in displacement, velocity, and acceleration in most cases. Compared to DSA, the improvement in displacement reduction ranged from 0.61 to 5.72%, with velocity reductions ranging from 2.04 to 6.77% and acceleration reductions ranging from 0.46 to 2.67%. For the 6-story building, the DSA algorithm outperformed the CryStAl algorithm in some cases. However, in 5 out of 6 cases, CryStAl still had the best performance among all the algorithms examined in terms of reducing displacement response. These remarkable improvements establish CryStAl as a high-performance algorithm for structural vibration suppression, outperforming the other examined algorithms.

It should be noted that CryStAl showed higher control force values compared to other algorithms, as indicated by index ${J}_{5}$. However, the overall performance of CryStAl in minimizing displacement and acceleration compensated this drawback. In this paper, the Friedman test was utilized to assess the efficiency of various algorithms used for vibration suppressions in two examples from a statistical point of view. As a result of this analysis, CryStAl demonstrated statistically significant reductions in displacement, velocity, and acceleration compared to the other algorithms for both building models.

The results demonstrate that the CryStAl algorithm is highly effective in controlling the response of both 3-story and 6-story shear model structures under seismic excitations. It delivers superior performance in reducing displacement and acceleration compared to the other optimization algorithms. The findings of this study indicate the potential of CryStAl and its applicability in the seismic control of structures. In summary, the following results were obtained through this study:

i. In most cases, the CryStAl algorithm outperformed PSO, SPO, HS, KH, ACO, GA, GWO, HHA, SSA, HO, and DSA in minimizing displacement and acceleration.
ii. The CryStAl algorithm consistently excelled in reducing displacement response across various earthquake scenarios for both the 3-story and 6-story buildings. In the 3-story case, CryStAl offered substantial improvements in comparison with the other algorithms, with average reductions of 17.58% and 3.51% compared to SPO and PSO, respectively. While the margins were smaller for HS, ACO, GA, GWO, HHO, SSA, HO, and DSA, the CryStAl algorithm still held a clear advantage over the other algorithms.
iii. For the 6-story building, CryStAl's dominance became even more apparent. Compared to SPO, it achieved an average reduction of 18.62%. Notably, CryStAl surpassed HS, KH, ACO, and SSA by margins exceeding 20%, showing substantial superiority for taller structures. Even against algorithms like GWO, HHO, HO, and DSA, where margins narrowed, CryStAl maintained a considerable lead.
iv. CryStAl's efficiency and effectiveness in reducing velocity and acceleration responses were validated, further establishing its position as a high-performance algorithm for structural vibration suppression. It also outperformed the other algorithms in terms of RMS top story displacement and acceleration.
v. The control force values in the algorithm were higher compared to almost all other algorithms, as indicated by index ${J}_{5}$. However, in the case of the 6-story building, there were some exceptions.
vi. The overall performance of CryStAl in minimizing displacement and acceleration outweighed its higher control force values. The findings highlighted the potential of CryStAl and its applicability to the seismic control of structures.
vii. This study compared various algorithms using a non-parametric test, revealing CryStAl’s superior performance in reducing vibrations across multiple response metrics (i.e., displacement, velocity, and acceleration) in both 3- and 6-story building models.

## Data Availability

The datasets used or analyzed during the current study are available from the corresponding author upon reasonable request.
